# Towards Augmented Human Memory: Retrieval-Induced Forgetting and Retrieval Practice in an Interactive, End-of-Day Review

**DOI:** 10.1037/xge0000441

**Published:** 2018-05

**Authors:** Caterina Cinel, Cathleen Cortis Mack, Geoff Ward

**Affiliations:** 1 Department of Psychology, University of Essex

**Keywords:** memory augmentation, retrieval-induced forgetting, wearable camera, smartphone, recall

## Abstract

The authors report 6 experiments that examined the contention that an end-of-day review could lead to augmentation in human memory. In Experiment 1, participants in the study phase were presented with a campus tour of different to-be-remembered objects in different university locations. Each to-be-remembered object was presented with an associated specific comment. Participants were then shown the location name and photographs of half of the objects from half of the locations, and they were asked to try to name the object and recall the associated comment specific to each item. Following a filled delay, participants were presented with the name of each campus location and were asked to free recall the to-be-remembered objects. Relative to the recall from the unpracticed location categories, participants recalled the names of significantly more objects that they practiced (retrieval practice) and significantly fewer unpracticed objects from the practiced locations (retrieval-induced forgetting, RIF). These findings were replicated in Experiment 2 using a campus scavenger hunt in which participants selected their own stimuli from experimenter’s categories. Following an examination of factors that maximized the effects of RIF and retrieval practice in the laboratory (Experiment 3), the authors applied these findings to the campus scavenger hunt task to create different retrieval practice schedules to maximize and minimize recall of items based on experimenter-selected (Experiment 4) and participant-selected items using both category-cued free recall (Experiment 5) and item-specific cues (Experiment 6). Their findings support the claim that an interactive, end-of-day review could lead to augmentation in human memory.

It is self-evident that we do not effectively encode all of the information that we encounter, nor can we retrieve all of the encoded content at will. Nevertheless, our accessibility to memories can be improved with effective cues, especially when those cues are specific and were presented originally at study (Nairne, 2002; Surprenant & Neath, 2009; Tulving, 1983). Technological advances can radically change the nature and the scale of the external cues that we can preserve to later probe recall: written diary entries can help us remember past daily events (Linton, 1975; Wagenaar, 1986), our photographs can help us relive our autobiographical experiences (Burt, Mitchell, Raggatt, Jones, & Cowan, 1995; Koutstaal, Schacter, Johnson, Angell, & Gross, 1998; Koutstaal, Schacter, Johnson, & Galluccio, 1999), and social media (Cosley, Sosik, Schultz, Peesapati, & Lee, 2012) can help remind us of shared events with family and friends. In this article, we consider the potential for lifelogging and smartphone technology to provide retrieval cues to augment human memory.

Our contention is that smartphone ownership is now so widespread (Miller, 2012; Smith, 2015) and the capabilities of lifelogging and wearable technologies are so advanced, that it is now possible to capture, store, and process multiple streams of near-continuous data, to be later used as cues to help retrieve associated episodic details (e.g., Bell & Gemmell, 2007, 2009; Clinch et al., 2016; Davies et al. 2015; Dodge & Kitchin, 2007; Gurrin, Smeaton, & Doherty, 2014; Harvey, Langheinrich, & Ward, 2016; Sellen & Whittaker, 2010; Silva, Pinho, Macedo, & Moulin, 2018). In particular, we were interested in the potential of an end-of-day memory review (e.g., Finley, Brewer, & Benjamin, 2011) to enhance the later recall of reviewed events through retrieval practice and also attenuate access to nonpresented but related events through retrieval-induced forgetting (RIF, Anderson, Bjork, & Bjork, 1994). To this end, we report data from six experiments that examined the mnemonic consequences of performing retrieval practice on a subset of digital images taken from earlier laboratory-based and real-world sequences of events.

There is already ample evidence that wearable cameras, such as SenseCam (e.g., Allé, Manning, Potheegadoo, Coutelle, Danion, & Berna, 2017; Hodges et al., 2006; Hodges, Berry, & Wood, 2011; Sellen, Fogg, Aitken, Hodges, Rother, & Wood, 2007; Silva, Pinho, Macedo, & Moulin, 2013) can enhance memory through an end-of-day review (Finley et al., 2011; Harvey et al., 2016). SenseCam is a small, neck-worn lifelogging device, so called because it can sense its environment and it records images using a built-in digital camera with a fish-eye lens. Central to its conception was the idea that the captured record of a digital event could be used as a memory cue to help reinstate further additional memories and details that were not currently accessible. By default, the camera takes time-stamped images every 30 s, but it can also be set to take photos given changes in sensor data (e.g., it can detect significant changes in light level, temperature, audio, movement, and body heat via passive infrared sensor levels). The camera can also be manually activated by the user and be switched off for moments of privacy. SenseCam was developed as a commercial device into the Vicon Revue and the Autographer (which incorporated GPS and Bluetooth technologies), and although these devices are not now commercially available, new products are now being marketed, which, among other features, also automatically records still images every 30 s.

From the earliest studies, SenseCam has been used to help with the rehabilitation of patients suffering from severe memory problems as well as healthy individuals. As reviewed by Silva et al. (2018), numerous case studies of patients (e.g., Berry et al., 2009; Doherty et al., 2012; Loveday & Conway, 2011; Pauly-Takacs, Moulin, & Estlin, 2011; Piasek, Irving, & Smeaton, 2012; Svanberg & Evans, 2014) with heterogeneous etiologies have consistently shown that SenseCam can be used to improve the retrieval of events depicted in the images relative to control baseline conditions or a personal diary. Memorial benefits for days in which SenseCam was worn and the images reviewed relative to control days have has also been shown with groups of young healthy participants, especially for recall (e.g., Finley et al., 2011; Sellen et al., 2007; Silva et al., 2013). However, in some studies that assessed recognition memory, SenseCam was also shown to increase false memories (St. Jacques & Schacter, 2013) and it may not help with the recognition of atypical experiences (Seamon et al., 2014). More importantly, Silva et al. (2018) question whether the review of SenseCam images provides the sense of recollection to associated details that is often assumed; in many studies recall following review increases, but it is uncertain whether or not the review can be used to elicit associated details that are not directly inferred from the images.

Early research on image review from wearable cameras was restricted by the limited availability of SenseCam and related products. Although the use of wearable cameras is not yet a mainstream activity, it is likely that the prevalence of wearable cameras may increase with the development of more recent products, such as the Narrative Clip, the GoPro, MeCam, the Google Clip, or through specialist Android phone applications (e.g., Hamm, Stone, Belkin, & Dennis, 2013; Nielson, Smith, Sreekumar, Dennis, & Sederberg, 2015; Sreekumar, Dennis, Doxas, Zhuang, & Belkin, 2014). There is now widespread ownership of smartphone devices (Miller, 2012; Smith, 2015) that are fitted with high quality cameras. There is also widespread practice of sharing one’s life activities through social media, and an increasing consumer appetite for other forms of lifestyle wearable technologies. Moreover, lifelogging and lifestyle smartphone apps are continually being developed, such that it is not completely far-fetched to imagine that one might routinely review the highlights of one’s own day via a smartphone app, in much the same way as one might be aware throughout the day of public news events via 24-hr news and media organizations, but nevertheless also read a summary digest of current news stories at the end of the day on a news app.

Reviewing, revising, rehearsing, and being tested on earlier episodes are likely to have mnemonic benefits to the later retrieval of these items (e.g., Anderson et al., 1994; Karpicke & Roediger, 2008; Roediger & Butler, 2011; Roediger & Karpicke, 2006a, 2006b; Roediger, Putnam, & Smith, 2011; Silva et al., 2018; Tan & Ward, 2000). However, an as yet unexplored issue within the SenseCam research community is whether the review of a subset of autobiographical images might lead to the attenuation in access to related memories through the phenomenon of RIF (Anderson et al., 1994).

For the original demonstration of RIF, Anderson et al. (1994) designed the retrieval practice paradigm consisting of three stages. In the initial study phase, participants were presented with 48 experimental category–exemplar pairs (such as Fruit–Orange or Tree–Hickory) for 5 s each, the stimuli comprising six exemplars from each of eight different semantic categories. In the subsequent retrieval practice phase, half of the exemplars from half the categories were tested on each of three occasions using the category name as the stimulus term and the first two letters of the exemplar as the specific cue for the response term (e.g., Fruit–Or____). Finally, in the test phase that was administered 20 min after retrieval practice, participants were cued with each of the category names and were asked to recall the studied exemplars. Anderson et al. found a recall advantage for the practiced exemplars: exemplars that had received retrieval practice, denoted *Rp+* items, were recalled significantly better than the baseline exemplars from those categories for which there was no retrieval practice, denoted *Nrp* items. However, Anderson et al. also observed that the recall of the nonpracticed exemplars from the practiced categories, the *Rp*− items, was significantly worse than the recall of the Nrp items, demonstrating that recall of these nonpracticed exemplars had been attenuated relative to baseline by the retrieval practice of other same-category exemplars.

The basic RIF finding appears robust, having been widely replicated in the laboratory using semantic category-exemplar pairs, and more recently using a variety of stimuli, scenarios, and tasks. For example, RIF has been studied in social cognition (Macrae & MacLeod, 1999), autobiographical memory (Barnier, Hung, & Conway, 2004; Ditta & Storm, 2016; Stone, Barnier, Sutton, & Hirst, 2013), socially shared and collective memory (Coman & Hirst, 2012; Coman, Manier, & Hirst, 2009; Cuc, Koppel, & Hirst, 2007), motor actions (Tempel, Aslan, & Frings, 2016; Tempel & Frings, 2013, 2014, 2015; Tempel, Loran, & Frings, 2015), eyewitness memory (Garcia-Bajos, Migueles, & Anderson, 2009; MacLeod, 2002; Shaw, Bjork, & Handal, 1995), second-language acquisition (Levy, McVeigh, Marful, & Anderson, 2007), using sentences (Gómez-Ariza, Lechuga, Pelegrina, & Bajo, 2005), factual knowledge (Anderson & Bell, 2001), advertisements (Parker & Dagnall, 2009), prose (Chan, 2009), and with visual, nonverbal material (Ciranni & Shimamura, 1999; Maxcey & Woodman, 2014). Moreover, Hicks and Starns (2004) have found evidence of RIF in item recognition.

Although the empirical finding of RIF appears robust, the theoretical interpretation of the RIF effect is highly contentious, with the most intense debate surrounding Anderson et al.’s claim that RIF stems (at least in part) from inhibition of the Rp− items. According to inhibition-based accounts of RIF (e.g., Anderson, 2003; Bäuml, 2007; Norman, Newman, & Detre, 2007; Storm & Levy, 2012), the retrieval practice of one category-exemplar pair should reduce the activation levels of all other competing items associated to the same cue (i.e., the inhibition of the Rp− items) through activation-reducing mechanisms (suppression). Anderson et al. (1994) acknowledged at an early stage that a noninhibitory account of RIF based solely on competition between differently activated exemplars could also explain this basic finding. According to a noninhibitory view, the accessibility of any particular item via a cue reflects the strength of the association of the cue to that item relative to the strength of association of that cue to all other competing items (e.g., Anderson, 1983; Mensink & Raaijmakers, 1988; Raaijmakers & Shiffrin, 1981; Rundus, 1973). If one assumes that each category cue was associated with six exemplar items in memory, then the retrieval practice performed on three of the exemplars serves to increase the association between the category cue and these respective practiced exemplars, which increases the relative strength of the practiced, Rp+ items and decreases the relative strength of the unpractised but related, Rp− items. Thus, these response-competition accounts offer a conventional way of explaining standard RIF effects without the need to assume inhibition.

Anderson (2003) proposed four properties of RIF that were argued to favor an inhibitory explanation of RIF over response competition accounts: cue-independence, retrieval specificity, interference-dependence, and strength independence. Evidence supporting the claim that an Rp− item itself is inhibited comes from the demonstration that retrieval practice reduces retrieval to inhibited items even from an independent cue (the cue independence property). Anderson and Spellman (1995) showed that retrieval practice of a category-exemplar pair, Red–Blood, not only reduced recall of nonpracticed experimental exemplars associated with Red (e.g., Red–Tomato) but, because of the pre-experimental association between, for example, Red and Strawberry, also reduced recall of similar related items that had been experimentally associated with an unpracticed category, for example, Food–Strawberry (see also Weller, Anderson, Gomez-Ariza, & Bajo, 2013). According to noninhibitory response competition accounts, retrieval practice on Red–Blood should not impair recall on Food–Strawberry, even if retrieval practice strengthens Red–Blood and weakens Red–Strawberry. Evidence supporting the claim that RIF occurs due to the suppression on Rp− items during an active retrieval attempt of competing Rp+ items (the retrieval specificity property) can be evidenced by the finding that simple re-exposure to studied items in the retrieval practice phase or other noncompetitive retrieval practice does not lead to RIF (Anderson, Bjork, & Bjork, 2000; see also Bäuml & Aslan, 2004). Evidence consistent with the claim that the magnitude of the RIF effect depends on the extent to which the Rp− items are competing with the Rp+ items (the interference dependence property) can be evidenced by the magnitude of the RIF effect being greater with stronger exemplars than weaker exemplars (Anderson et al., 1994). Finally, the claim that the magnitude of the RIF effect need not be directly related to the extent to which Rp+ items have been strengthened (the assumption) can be supported by the finding that RIF can occur even when no competitor is strengthened (Storm, Bjork, Bjork, & Nestojko, 2006).

For our purposes, we were primarily interested in whether or not we would obtain retrieval practice and RIF with autobiographical images and were less interested in discerning between different theoretical accounts. This is fortunate because a very large number of laboratory studies have been conducted using variants of the standard retrieval practice paradigm in order to seek greater theoretical insight and support for different RIF mechanisms, and different articles and reviews of this literature are either more persuaded (Anderson & Levy, 2007; Bäuml, 2007; Kliegl & Bäuml, 2016; Levy & Anderson, 2002; Murayama, Miyatsu, Buchli, & Storm, 2014; Storm et al., 2015) or less persuaded (Perfect et al., 2004; Raaijmakers & Jakab, 2013; Verde, 2012; Williams & Zacks, 2001) for the need for inhibition. Moreover, a more recent, alternative (noninhibitory) theory based on changes in context (Jonker, Seli, & MacLeod, 2013) argues that the retrieval practice paradigm produces RIF because the context at test more closely matches the context at retrieval practice than the context at original study. Jonker et al. found that RIF could be eliminated following manipulations at test that helped reinstate the original study context (a claim that has itself already attracted some counterclaims, see Chan, Erdman, & Davis, 2015; Soares, Polack, & Miller, 2016).

Even if we concern ourselves less with the theoretical interpretation of RIF, there are still a number of issues that make it far from certain as to whether or not we will obtain RIF with images of autobiographical stimuli. If one were to design a study to maximize the chances of observing RIF, one might use strong exemplars (Anderson et al., 1994) that are not highly integrated with each other (Anderson & McCulloch, 1999; Chan, 2009) from discrete, nonoverlapping categories. By contrast, in real life, there is likely to be a large variation in the strength of associations between the photographic images related to a higher-order event, and the exemplar images are quite likely to be associated with each other (i.e., integrated) and each event is likely to be associated with multiple overlapping event categories. Nevertheless, recent evidence supports the possibility of retrieval practice and RIF using other forms of visual stimuli using recall, such as sets of slides and video (e.g., Migueles & Garcia-Bajos, 2007; Shaw et al., 1995), and digital images (Koutstaal et al., 1999) as well as recognition-induced forgetting in the recognition of visual objects (Maxcey & Woodman, 2014; Maxcey, 2016).

Therefore, prior research suggests that it may be theoretically possible that, in the near future, captured images of our day might be used to augment the future recall of selected events and attenuate related but unreviewed material. Any such attenuation may be unwanted (one might not wish to attenuate access to unreviewed holiday memories via selective review of a subset). However, if the RIF finding is observed, it would be important for users of the technology to be informed of this phenomenon, and to offer empirical data quantifying the magnitude and the extent of these effects, so that there could be evidence-based guidance provided on the degree of loss that could be expected. Alternatively, it could be that in the near future, a technological user may actively wish to attenuate related but unreviewed material, perhaps with the direct aim of reducing the accessibility to out-dated or unwanted memories.

In the remainder of this article, we report six experiments that directly addressed whether retrieval practice and RIF effects could be observed using a subset of digital images related to prior events. In Experiment 1, we examined retrieval practice and RIF effects using experimental stimuli that were selected and presented by the experimenter during a campus tour of different university locations. To anticipate our findings, we found that the later recall of tour objects could be heightened (retrieval practice) or attenuated (RIF) following a retrieval practice review phase. In Experiment 2, we used a campus scavenger hunt to show that these retrieval practice and RIF effects could be extended to include to-be remembered items that were selected by the participant rather than the experimenter. In Experiment 3, we examined in the laboratory three different manipulations that we thought had the potential to affect the magnitude of retrieval practice and RIF effects. These laboratory studies informed our use of different retrieval practice schedules on a further campus scavenger hunt experiment (Experiment 4) to maximize and minimize later recall. We then developed a basic user-interface to allow participants in a scavenger hunt experiment (Experiment 5) to select which of their participant-generated stimuli they wished to remember and which they wished to forget through the retrieval practice phase. We observed improved recall for items selected to be remembered, and impaired recall for items selected to be forgotten. These findings were observed using tests of free recall using a category cue (Experiment 5) and tests of cued recall using item-specific probes (Experiment 6).

## Experiment 1

In Experiment 1, we sought to examine the effects of retrieval practice and RIF of personally experienced events in the real world, using a method that was loosely based on the laboratory retrieval practice paradigm (Anderson et al., 1994). The study phase consisted of a guided tour of the university campus, in which small groups of between one and four individuals were taken to eight different experimental locations on campus, and in each location, six objects would be identified as a to-be-remembered items. For each to-be-remembered item they would be told an associated comment that was specific to that object.

After the campus tour, half of the participants (those in the retrieval practice group) took part in a retrieval practice phase in which they were presented with images of half the to-be-remembered items that they had experienced in half the campus locations. For each image, participants were asked to try to think back to the original study experience and attempt to name the object and retrieve the associated comment that was specific to that item. Finally, following an unrelated filler task, participants were presented with the names of the different campus locations and for each location they were asked to free recall the to-be-remembered objects that had been identified in that location.

A second group of participants in the no retrieval practice group received no retrieval practice. This additional control group was designed to allow comparison of recall on the Nrp items across the two groups. It is often assumed that the items from unpracticed categories are unaffected by the retrieval practice performed on other categories, and while this may be the case for clearly defined semantic categories, we thought that it would be prudent to consider whether this was also the case for autobiographical memories.

Finally, an unrelated purpose of the experiment was to investigate camera usage and user experience of four different capturing devices during the tour. There has been the suggestion that actively capturing images may decrease participants’ reliance on their own memories (e.g., Henkel, 2014). To this end, and orthogonal to our investigations into the effects of retrieval practice, we provided one quarter of the participants with a wearable camera, a narrative clip, which automatically takes a photograph every 30 s; we provided one quarter of the participants with a smartphone and encouraged them to take as many photographs as they wished during the tour using the standard smartphone camera application, we provided one quarter of the participants with a smartphone camera using a special Android application, My Good Old Kodak (Niforatos, Langheinrich, & Bexheti, 2014), which allowed them to take a total of 24 photographs during the tour; and for one quarter of the participants we provided no technology.

### Method

#### Participants

A total of 80 students from the University of Essex participated in this experiment in exchange for either a small payment or course credit.^1^ The experiment was approved by the research ethics committee of the University of Essex.

#### Design

The experiment used a 2 × 4 × 3 mixed design. The first between-subjects independent variable was review condition with two levels (retrieval practice group and the no retrieval practice group). The second between-subjects independent variable was technology group, with four levels such that there was a no device group, a My Good Old Kodak group, a smartphone group, and a narrative clip group. Within the retrieval practice group, there was a single within-subjects independent variable, retrieval practice status, with three levels: items that were practiced during the retrieval practice phase (Rp+); items that were not practiced but were from the same campus tour locations as the practiced items (Rp−); and items that were not practiced and did not belong to any of the Rp+ item locations (Nrp). The dependent variable was the proportion of to-be-remembered items from each campus tour location that were correctly recalled.

#### Material and apparatus

For the study phase, a total of 10 different locations on the University of Essex campus were selected. These were the Biology Building, the Car Park, the Garden, the Lake, the Library, the Podia, the Sports Center, Square 4, the Student Union, and the Teaching Centre. At each of the 10 locations, we selected six to-be-remembered items, and we invented an additional, item-specific comment. These comments were plausible but not necessarily true and went beyond merely pointing out a perceptual feature of the object (e.g., Library- Printer: “This is the most used printer in the library”; Lake–Bench: “There are 72 identical benches on campus”). A full list of locations, to-be-remembered objects, and their associated comments are presented in Appendix A. The set of 60 images are available as online supplemental material associated with this article. Of the 10 locations, two were chosen as filler locations (Square 4 and the Student Union) and these were visited as the first and the last location of each tour. The remaining eight locations were used as the experimental categories. A large sheet of paper with the location, the to-be-remembered object and the associated comment was generated for each stimulus item, to be presented in situ to reinforce the identity of each of the experimental stimuli.

For the retrieval practice phase, stock photographs of each of the to-be-remembered items were taken and sized at 1,024 × 768 pixels. These stock images would be presented to participants in the laboratory while undertaking retrieval practice in the retrieval practice phase. Retrieval practice was recorded via the Audacity 2.0.5 application. The retrieval practice phase and the recall phase of the experiment were presented using the SuperCard 4.7 application of the Apple Macintosh computer, and responses for the recall phase were written down on the provided response sheets (one for each location).

Participants who were allocated to a Technology group were equipped with either a narrative clip (measuring only 36 × 36 × 9 mm and weighing only 20 g, which is simply clipped onto the participants’ clothing) or a Samsung Galaxy SIII Mini smartphone. Participants in the smartphone group used the smartphone’s default camera application (and the participant could therefore take unlimited images), whereas participants in the My Good Old Kodak group also used the smartphone camera, but used the Android application of the same name (which limited the number of photographs that could be taken to 24, like a traditional Kodak 35 mm camera), downloaded from the Google Play Store.

#### Procedure

Participants were assigned to one of four Technology groups: the no device group, the My Good Old Kodak group, the smartphone group, and the narrative clip group (20 participants per group). Half of participants within each technology group were allocated to the retrieval practice group, and the remaining participants within each technology group were assigned to the no retrieval practice group. Participants issued with cameras were told how the technology worked, the numbers of photos available, and they were encouraged to take photos during the campus tour.

All participants were then given a guided tour of the university campus tour in tour subgroups of between 1 and 4 people and within each tour subgroup, all participants were in the same technology condition. There were 6 different experimental routes through the campus, and each route visited the locations in different orders. Although the presentation order of the to-be-remembered items at each location was completely randomized, it was impractical to completely randomize the tour locations. At each campus location, participants were informed of the location name. Then, for each of the six to-be-remembered items of a location, the experimenter pointed to the item, named the item, and said out loud the specific comment associated with the item. To ensure that all participants in the subgroup had paid attention and had a fair opportunity to encode each item and comment, a sheet of paper detailing the location, the name of the item, and the associated comment of each item was also shown to the participants during encoding. The sheet of paper also acted as an aide memoire to the experimenter conducting the tour.

Once the campus tour was completed, the participants were asked to attend a session in the laboratory. Participants who were assigned to the no retrieval practice group were asked to sit at a computer and were told they were free to browse the Internet. This was done as a filled interval, while the other participants, who were assigned to the retrieval practice group, took part in the retrieval practice session. These participants were presented with three of the six stock images from one of the filler locations and from four of the eight experimental locations. Each stock image was presented for 10 s, together with the name of its location and the first two-letters as a cue. Thus, they may see a stock image of the printer in the library, with the text: “Library Pr___” and the participants’ task was to recall aloud the item and the associated item-specific comment that they had been told during the study phase. Once the retrieval practice phase was completed, all participants performed a set of unrelated semantic fluency filler tasks, in which participants tried to recall as many exemplars as possible from each of 8 semantic categories. Participants were given 60 s per category to perform these fluency tasks.

The final test phase of the experiment was a final recall test. Participants were presented with the name of a campus location and they were given 30 s to write down as many of the to-be-remembered items as they could from that campus location. All participants were first assigned a filler location, followed by the eight experimental locations in a random order. Participants were free to recall the items at each location in any order.

To assess the participants’ experiences of using the different types of technology, and to assess the subjective qualities associated with their captured images, all participants were invited back after 1 week, and their delayed memory for the campus tour was assessed before and after viewing the photographic images that they had personally captured with the provided technology. The users’ review and experiences of the different technologies are reported elsewhere (Niforatos, Cinel, Mack, Langheinrich, & Ward, 2017), but we took advantage of testing all the participants immediately upon their return on a delayed final memory test, using exactly the same procedure as the final recall test.

### Results

Table 1 shows the critical data for the proportion of correctly recalled items by retrieval practice status for the retrieval practice group in the original and delayed recall tests. The more detailed recall from each of the different technology conditions can be found in Appendix B.[Table-anchor tbl1]

#### Recall in the test phase

The primary purpose of the experiment was to examine the effect of retrieval practice upon the recall in the test phase for the participants in the retrieval practice group. A 4 (technology: no technology, My Good Old Kodak, smartphone, narrative clip) × 3 (retrieval practice status: Nrp, Rp+, Rp−) analysis of variance (ANOVA) was performed on the proportion of items recalled in the test phase. This revealed a significant main effect of retrieval practice status, *F*(2, 72) = 59.1, *MSE* = .013, η_p_^2^ = .621, *p* < .001; a nonsignificant main effect of technology, *F*(3, 36) = 1.58, *MSE* = .051, η_p_^2^ = .116, *p* = .212;and a nonsignificant interaction, *F*(6, 72) = .780, *MSE* = .013, η_p_^2^ = .061, *p* = .589.

A paired *t* test showed that the recall of Rp+ items (*M* = .798) was significantly greater than the recall of the Nrp items (*M* = .597), confirming significant positive effect of retrieval practice, *t*(39) = 8.12, *p* < .001. A second paired *t* test showed that the recall of Rp− items (*M* = .529) was significantly reduced relative to the recall of the Nrp items (*M* = .597), indicating that there was significant retrieval induced forgetting, *t*(39) = 2.94, *p* = .006.

Finally, it is often assumed using laboratory stimuli that retrieval practice within certain categories has little or no effect on the recall of other categories. In keeping with this assumption, we found no significant difference between the recall of the Nrp items in the no retrieval practice group items (*M* = .635) and the Nrp items in the retrieval practice group (*M* = .597), *t*(78) = 1.18, *p* = .240.

#### Delayed memory test

A 4 (technology: no technology, My Good Old Kodak, smartphone, narrative clip) × 3 (retrieval practice status: Nrp, Rp+, Rp−) ANOVA was also performed on the proportion of items recalled given the location cues in the delayed test taken 7 days after the campus tour. This revealed a significant main effect of retrieval practice status, *F*(2, 72) = 31.3, *MSE* = .024, η_p_^2^ = .465, *p* < .001; a nonsignificant main effect of technology, *F*(3, 36) = .0.790, *MSE* = .087, η_p_^2^ = .062, *p* = .508; and a nonsignificant interaction, *F*(6, 72) = .991, *MSE* = .024, η_p_^2^ = .076, *p* = .438.

A paired *t* test showed that the delayed recall of the Rp+ items (*M* = .658) was significantly greater than the delayed recall of the Nrp items (*M* = .441), *t*(39) = 5.95, *p* < .001, indicating sustained positive mnemonic benefits from retrieval practice over a 7-day retention interval. A second paired *t* test showed that the delayed recall of the Rp− items (*M* = .404) was not significantly different from the delayed recall of the Nrp items (*M* = .441), *t*(39) = 1.16, *p* = .255, indicating that the RIF effect was no longer significant following the delay.

### Discussion

Experiment 1 found significant retrieval practice and RIF effects for real world objects in a real-world scenario (an Experimenter-guided campus tour). Specifically, when participants were given a campus tour and then shown images of half of the to-be-remembered objects from half of the campus locations, the requirement to try to recollect the associated comments to each object led to the subsequent increase in later recall of the practiced items (significant retrieval practice effects) and the subsequent reduction in later recall of the unreviewed objects from the related locations (significant RIF effect), relative to the recall of items from unreviewed locations.

This set of findings strongly suggest that both retrieval practice and RIF effects can be elicited in the real world through the review of captured data using smartphone or lifelogging digital technologies. Although earlier and more recent evidence supports the possibility of retrieval practice and RIF using sets of slides and video (e.g., Migueles & Garcia-Bajos, 2007; Shaw et al., 1995), digital images (Koutstaal, Schacter, Johnson, & Galluccio, 1999) as well as in the recognition of visual objects (Maxcey & Woodman, 2014; Maxcey, 2016), there were a number of reasons why we might question whether we would obtain RIF effects in this experiment. First, the strongest evidence for RIF effects has been when using strong exemplars (Anderson et al., 1994) that are not highly integrated with each other (Anderson & McCulloch, 1999; Chan, 2009) from discrete, nonoverlapping categories. By contrast, in our experiment there were likely to have been a large variation in the strength of associations between the photographic images of to-be-remembered objects and their locations, and the objects were quite likely to be associated with each other (i.e., integrated) and some objects (e.g., bench), were likely to be associated with multiple overlapping locations. Such variation in associations and the overlap between events is likely to be even more pronounced in real life events, and so it is at least promising that basic retrieval practice and RIF effects can occur using this methodology.

However, although there was clear evidence of retrieval practice and RIF effects in the original recall test after only a short delay (approximately 15 min), the effects of RIF had dissipated after an extended delay of 1 week. This is consistent with some other studies suggesting that RIF effects are sometimes eliminated after a 24-hr delay (e.g., Chan, 2009; MacLeod & Macrae, 2001) but it should be noted that there are instances of RIF persisting a number of days (e.g., Garcia-Bajos et al., 2009; Migueles & García-Bajos, 2007; Storm, Bjork, & Bjork, 2012; Storm et al., 2006).

A third finding, unrelated to the primary purpose of this experiment concerned the role of technology on later memory. Although participants who were not given any technology numerically outperformed those who were given a photographic device (Henkel, 2014), there were no significant differences in participants’ recall of test items between any of the four technology groups used during the campus tour.

## Experiment 2

Experiment 2 used the campus scavenger hunt method to examine whether it was possible to generate retrieval practice and RIF effects when the to-be-remembered exemplars were generated by the participants themselves. When one considers the material and tasks for which retrieval practice and RIF have been observed, the vast majority of those studies have been set in the laboratory using semantic category-exemplar stimuli (e.g., Anderson et al., 1994), and when more real-world stimuli have been used (such as in Experiment 1), they are normally generated and presented by an experimenter (e.g., Shaw et al., 1995), or the methods have involved the retrieval of events that occurred prior to the experimental study (e.g., Barnier et al., 2004). For the full potential of using lifelogging technologies in an end-of-day review, it is essential that we examine whether it is possible to obtain both retrieval practice and RIF effects using participant-generated stimuli in which we have an objective account of their encoding.

To this end, we developed the campus scavenger hunt method, in which participants were given a list of 10 categories (eight experimental categories and two filler categories), and for each category, participants were asked to find six different exemplars around the university campus and take a photograph of each exemplar. The experimenter-generated categories were either conceptual (e.g., “something electrical,” “something living,” “something made of metal, “something edible”) or perceptual (e.g., “something orange,” “something with stripes,” “something soft,” “something circular”).

The participants were provided with an Android smartphone on which was installed the application, My Good Old Kodak (Niforatos et al., 2014), which was used by participants to take digital photographs of their chosen objects. The primary advantage of this application is that users of the application cannot see the photographs that they take, such that our participants were not able to revise, rehearse, or review photographs of their objects during the study phase. A secondary advantage is that the My Good Old Kodak application limits the number of photographs that are remaining on a camera roll, and limits the availability of camera rolls, such that we could require participants to take a certain number of objects of a particular category, before moving onto the next category. The My Good Old Kodak application displays the number of remaining photos that are left on that camera roll, and so the application allows the user to keep track of the number of exemplars yet to be selected. When taking the photographs, participants were also asked to think about an idiosyncratic comment associated with each exemplar, which they spoke into the audio microphone and was recorded by the smartphone.

After completing the campus scavenger hunt task, participants’ digital images were downloaded from their smartphones. During the retrieval practice phase, they were presented with the category names and the images of half of the participant-selected objects from half of the experimenter-provided categories. The participants were requested to name the participant-selected object and recall the associated comment that they had recorded. In the final test, the participants’ memory of the to-be-remembered objects was then tested with a category-cued test of free recall.

### Method

#### Participants

A total of 40 students from the University of Essex participated in this experiment in exchange for either course credit or £15. The experiment was approved by the research ethics committee of the University of Essex.

#### Design

There was one within-subjects independent variable, the retrieval practice status, with three levels: items that were practiced during the retrieval practice phase (Rp+); items that were not practiced but were from the same categories as the practiced items (Rp−); and items that were not practiced and did not belong to any of the practiced categories (no retrieval practice, Nrp). The dependent variable was the proportion of items that were correctly recalled in the memory test.

#### Stimuli and apparatus

There were eight experimental categories: “something electrical,” “something made of metal,” “something orange,” “something with stripes,” “something living,” “something soft,” “something circular,” and “something edible”; and two filler categories (“something with writing” and “something bigger than a dog”). The exemplars for each category were not determined by the experimenter but were selected by the participants. Participants were equipped with a Samsung Galaxy SIII Mini smartphone and used the My Good Old Kodak Android application (Niforatos et al., 2014) which was downloaded from the Google Play store to capture the images, and the smartphones’ voice recorder to record the participants’ comments. Participants’ verbal responses during retrieval practice were recorded with Audacity 2.0.5. The experiment and instructions in the retrieval practice and test phase were presented using the SuperCard 4.7 application on an Apple Macintosh computer.

#### Procedure

The experiment was divided into two sessions. The first session (which included the study phase) was completed in the morning, while the second session took place in the laboratory in the afternoon of the same day, during which participants completed a retrieval practice task, a filler task and finally a memory test. The gap between the end of the first session and the beginning of the second session was of at least two hours.

In the morning session, participants were given a smartphone and shown how to use the smartphone’s voice recorder and the My Good Old Kodak application. This application had been set to deliver sets of camera rolls, each of six shots. The participants could always see the number of shots that were remaining on the roll (e.g., 2/6) on the viewfinder. The participants were also given a printed list with 10 experimenter-selected categories. The list always started and ended with a filler category, but the orders of the eight experimental categories were randomized for each participant.

The participants were instructed to take photographs of six different items that matched each of the 10 categories. For each captured item, participants were instructed to record an interesting or memorable fact or associated comment using the phone’s voice recording app. Participants were told that the comment could be anything that they associated with the item, or some peculiar details about it or its context, but must not be simply a statement about the identity of the item. Participants were told that these comments would need to be retrieved in the afternoon session. Participants were also instructed to follow the order of the categories on the printed list, and to take all six photos within a particular category before taking photos of the next categories.

The digital images taken with the My Good Old Kodak application could not be viewed immediately by participants but were automatically stored within a hidden folder on the mobile phone. A maximum of 2 hr were allocated for the first session, and photos could be captured anywhere on the University of Essex campus. Once participants completed the first session, all their captured digital images and voice recordings were downloaded from the phone. All digital images were checked to ensure that they matched the respective category.

In the afternoon session, participants completed a standard retrieval practice phase (Anderson et al., 1994), during which they were shown half of the six digital images from half of the eight experimental categories, together with three items from one of the filler categories. The 15 selected digital images were each presented three times, the order of the set of 15 images was randomized separately on each of the three presentations. Each digital image was presented along with the name of its category for 10 s, and participants were required to name out loud the item in the digital image as well as say the specific associated comment that they had said during the first session when they captured that photo.

The retrieval practice was followed by a filler task, in which participants were shown the names of eight different European countries (France, Spain, Italy, Germany, Greece, Holland, Ireland, Austria, Turkey, and Russia). The country names were displayed one at a time, in a randomized order. The participants were given 1 min per country to write down as many tourist activities and attractions that they might do or see if they went on holiday to each of the countries. The purpose of the filler task was to create a time interval between the retrieval practice and a final memory test, while engaging participants, during that interval, on an activity that was unrelated to the experimental stimuli.

Finally, participants had to complete a memory test, during which they were cued with the category names (e.g., “Something orange”), which were presented one at a time, and for each, they were required to write down as many items from that category as they could remember. They were given 30 s per category to write down the name of the items or a short description of the image/item. The first cued category was always one of the filler categories, followed by the eight experimental categories in a randomized order. Participants were free to write down the recalled objects in any order that they wished.

We wanted to check that the items recalled by participants in the memory test items had actually been captured. For this reason, the experimenter scored the memory test at the end of the session in the presence of each participant. For each category, the participant was shown a display with the six digital images that he or she had taken and was asked to use these to crosscheck against the recalled items that they had written down in the memory test sheet for that specific category.

### Results

The main findings from the final recall test of Experiment 2 are shown in Table 1. As can be seen, accuracy was higher for Rp+ items (*M* = .798) than for Rp− items (*M* = .513) and Nrp items (*M* = .658). A within-subjects ANOVA showed that there was a significant main effect of retrieval practice status, *F*(2, 78) = 56.4, *MSE* = .014, η_p_^2^ = .591, *p* < .001. Paired *t* tests showed that Rp+ items were recalled significantly better than Nrp items, *t*(39) = 6.21, *p* < .001, and Rp− items, *t*(39) = 9.08, *p* < .001, showing retrieval practice effects. In addition, the Rp− items were recalled significantly less often than Nrp items, *t*(39) = 5.61, *p* < .001, demonstrating significant RIF effects.

### Discussion

The results from Experiment 2 replicated and extended the findings of Experiment 1 and confirmed that retrieval practice and RIF could be observed with episodic memories of events experienced in the real world. Previous demonstrations of these effects with autobiographical memory have typically used stimuli that were either presented to the participants (e.g., Experiment 1; Shaw et al., 1995) or were studied prior to the experiment (e.g., Barnier et al., 2004; Stone et al., 2013). By contrast, in this experiment we have demonstrated these same effects with objects that were encoded during the experiment (for which we have objective evidence) and that were all chosen by the participants.

The discovery of retrieval practice and RIF effects with participant-generated stimuli further strengthens the possibility that lifelogging technology could be used to both facilitate and attenuate later accessibility to experienced events. We envisaged that an “end-of-day” review (Finley et al., 2011) could provide highlights of one’s day, and that different schedules presented at that review, might facilitate or even attenuate accessibility to events that were desired to be more or even less accessible. Having confirmed that RIF can indeed occur in real-life settings, our next step was to determine whether we could manipulate the magnitude of this facilitation and attenuation to maximize the likelihood of remembering—or even forgetting—specific memories.

## Experiment 3

In Experiment 3, we examined three different factors that we thought might affect the degree of retrieval practice and RIF. To do this, we used the standard laboratory-based methodology using classic semantic stimuli, before applying our discoveries in more real-world situations in Experiments 4, 5, and 6.

**Figure 1 fig1:**
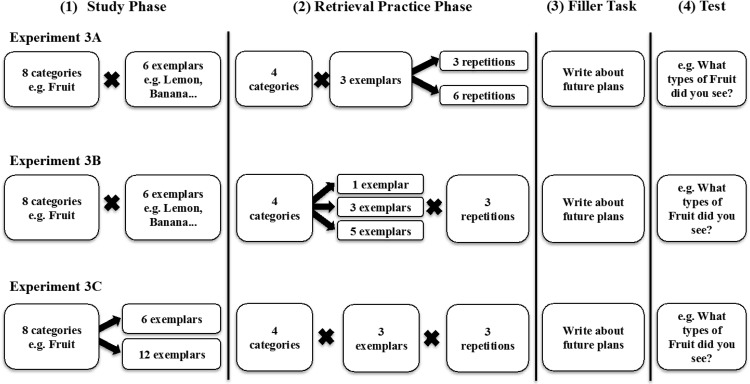
A schematic diagram of the procedure of Experiments 3A, 3B, and 3C.

The three factors that we examined in Experiment 3 can be summarized in Figure 1. It depicts a skeletal diagram of the “standard” Anderson et al. (1994) laboratory-based retrieval practice methodology. Typically, participants Study 6 exemplars from each of eight different experimental categories, and then receive three repetitions each of half of the exemplars from half of the categories. As Figure 1 shows, we manipulated the number of repetitions of each cue in the retrieval practice phase from three to six (Experiment 3A), the number of exemplars that were practiced in a standard category from one, three, or five exemplars out of six (Experiment 3B), and the number of exemplars studied within a category from six to 12 exemplars (Experiment 3C).[Fig-anchor fig1]

In Experiment 3A, we examined the extent to which later accessibility was affected by the number of times each cue was presented during the retrieval practice phase, and we manipulated the number of repetitions from between three and six. We assumed that increasing the number of repetitions of retrieval practice would increase the facilitation to those multiply represented items. The effect of the number of repetitions of practiced (Rp+) items in retrieval practice has been investigated previously by Macrae and MacLeod (1999, Experiment 3), who presented the Rp+ cues one, three, and six times. Using social cognition stimuli, participants were told that they had to “form impressions” of two men, John and Bill. They were presented at study with descriptions of each man containing 10 traits. Half of the traits of one man were presented one, three or six times prior to a final recall test. They found only a marginal effect on the degree of retrieval practice and no effect on the RIF. By contrast, no effect of the number of repetitions of practiced items was reported by Maxcey (2016) using “recognition-induced forgetting” which used visual stimuli and a procedure that deviates significantly from the standard method.

The second factor that we thought might affect the degree of retrieval practice and RIF was the number of exemplars from a particular category that were cued in the retrieval practice phase. Using the principles of encoding specificity (Tulving & Thomson, 1973; Tulving, 1983) and cue overload (Surprenant & Neath, 2009; Watkins & Watkins, 1976), we predicted that the heightened accessibility of a practiced exemplar might be affected by the specificity of the category cue: as the number of practiced exemplars from a category was increased, so the increase in any one particular practiced item might decrease due to cue overload. However, we also thought that increasing the number of practiced exemplars within a category might increase the degree of RIF. If the retrieval accessibility of related but unpracticed items was decreased either by increased competition (e.g., Rundus, 1973) or by increased inhibition (Anderson, 2003) by every related item that was practiced, one might expect that as the number of practiced exemplars from a category was increased, so there would be increasing magnitude of RIF, resulting in reduced recall of the related but unpracticed items. Thus, in Experiment 3B, we manipulated the number of practiced exemplars within a category from one, three, or five practiced exemplars per practiced category.

The final factor that we manipulated was the number of exemplars in the category. In the standard method, participants are presented with 6 exemplars from each category. In Experiment 3C, we contrasted the retrieval practice in categories of six and 12 exemplar items. Although it is not immediately clear how real-world items would be categorized, it is highly likely that certain related events are more regular than others, leading to a wide variation in functional category sizes. One might imagine that the degree of inhibition (or increased competition) of a related item as a result of retrieval practice might be reduced as the category set size increases (e.g., due to the likely decrease in average taxonomic strength in larger sets).

## Experiment 3A

In Experiment 3A, we used the standard retrieval practice methodology but manipulated the number of repetitions from three to six given to each cue in the retrieval practice phase.

### Method

#### Participants

Eighty students from the University of Essex were paid £5 in return for participation. The experiment was approved by the research ethics committee of the University of Essex.

#### Design

We used a 2 × 3 mixed design, where the between-subjects factor was the number of cue repetitions (with two levels: three repetitions and six repetitions), and the within-subject factor was the retrieval practice status of each item (with three levels: Rp+, Rp−, and Nrp). The dependent variable was the proportion of items recalled in the final category-cued memory test.

#### Material and apparatus

We used eight experimental categories (“Fruits,” “Leather,” “Trees,” “Weapons,” “Professions,” “Drinks,” “Hobbies,” and “Metals”) and two filler categories (“Fish” and “Insects”). The eight experimental categories were the same as those used by Anderson et al. (1994) in their Experiments 1 and 2. Six items were selected for each category. However, we did not use exactly the same exemplars as those used by Anderson et al. (1994), but rather based our exemplar selection on the exemplars generated from 40 different participants. The complete set of stimuli that was used is shown in Appendix C. The experiment and instructions were presented using the application, SuperCard 4.7, via an Apple Macintosh computer. In the second phase of the experiment, participants’ verbal responses were recorded with the Audacity 2.0.5 application.

#### Procedure

The experiment followed the standard retrieval practice methodology. In the study phase, participants were presented with a series of Category - Exemplar pairs (e.g., “Tree–Willow”). Each exemplar was shown for 5 s and participants were instructed to look at each category-exemplar pair and try to think about how these pairs of words related to each other. In the study phase, each participant was presented with all the exemplars from all 10 categories, in randomized order, with two restrictions: (a) that, for each item, one item from each of the 10 categories had to be presented before another item from the same category was presented, and (b) that the first two and last two items of the study phase had to be filler items.

The study phase was immediately followed by the retrieval practice phase. In this phase, three exemplars from each of four experimental categories (together with three exemplars from one of the two filler categories) were represented to participants for retrieval practice. The selection of exemplars and categories was randomized. Retrieval cues associated with each of the 15 selected items were presented for 10 s, in a random order, and each cue consisted of the category name and the first two letter of the exemplar name (e.g., Tree–Wi______). Each retrieval cue was repeated either three or six times, depending on the experimental condition. In each repetition of the cues, the cues were presented in a different random order, the only restrictions being that the first two and last two items were fillers. Participants were instructed to say out loud the cued word (if they could remember it), and their voice was recorded. In the filler task, participants were asked to describe, in words and/or drawings, how they represent things that they plan to do in the future (e.g., appointments, planned activities, things to do, etc.).

In the final memory test, participants were shown the name of one filler category and all eight experimental categories and were asked to write down, for each category, as many of the exemplars that were paired with that category (at any stage of the experiment) as they could remember, in any order that they wished. They were given 30 s per category. The first category was always the filler category and then all the experimental categories were presented in a random order.

### Results and Discussion

Table 1 shows the mean proportion of exemplars recalled in the final category-cued recall task of Experiment 3A. The data are separated by the retrieval practice status of the exemplar, and whether the practiced items had each been practiced 3 or 6 times. A 2 × 3 mixed ANOVA was performed with number of cue repetitions as the between-subjects factor with 2 levels (three repetitions group, and six repetitions group), and retrieval practice status as a within-subject variable with three levels (Rp+, Rp−, Nrp). There was a significant main effect of retrieval practice status, *F*(2, 156) = 242.0, *MSE* = .015, η_p_^2^ = .756, *p* < .001; a nonsignificant main effect of number of cue repetitions, *F*(1, 78) = .157, *MSE* = .053, η_p_^2^ = .002, *p* = .693; and a significant interaction, *F*(2, 156) = 5.83, *MSE* = .015, η_p_^2^ = .070, *p* = .004.

In examining the interaction, we first examined the effects of retrieval practice and RIF separately for the two different groups. Considering first the recall in the three repetitions group, paired *t* tests showed that Rp+ items were recalled significantly more often than Nrp items, *t*(39) = 10.6, *p* < .001, and Rp− items were recalled significantly less often than Nrp items, *t*(39) = 2.24, *p* < .05, showing significant retrieval practice and RIF effects. Considering next the recall in the six repetitions group, paired *t* tests showed that Rp+ items were recalled significantly more often than Nrp items, *t*(39) = 12.9, *p* < .001, and Rp− items were recalled significantly less often than Nrp items, *t*(39) = 3.18, *p* < .01, also showing significant RIF effects.

To further explore the interaction, we then calculated for each group the benefit in recall through retrieval practice by subtracting the Nrp values from the Rp+ values and the reduction in recall through RIF by subtracting the Rp− values from the Nrp values. An independent samples *t* test showed that there was greater benefit to recall of retrieval practice in the six repetitions group (+.375) than in the three repetitions group (+.279), *t*(78) = 2.45, *p* = .017. However, a further independent samples *t* test showed that there was no significant difference between the reduction in recall due to RIF in the six repetitions group (.052) than the reduction in recall due to RIF in the three repetitions group (.081), *t*(78) = 0.843, *p* = .402.

Our analyses therefore indicated that there were clear retrieval practice and RIF effects in both groups, but whereas we found evidence that practiced items benefit from further repetitions, we did not find evidence that multiple repetitions led to significantly stronger RIF effects. These findings are consistent with some previous results (Macrae & MacLeod, 1999) but for alternative findings, see Parker and Dagnall (2009).

## Experiment 3B

In Experiment 3B, we again used the standard retrieval practice methodology but manipulated the number of exemplars that were practiced. Specifically, different groups received retrieva1 practice on one, three, or five (out of six) exemplars from half of the categories in the retrieval practice phase. All practiced items received three repetitions of practice. Categories and stimuli were identical to those used in Experiment 3A.

### Method

#### Participants

A total of 120 students from the University of Essex were paid £5 in return for participation. They were allocated upon arrival to one of three separate groups. The experiment was approved by the research ethics committee of the University of Essex.

#### Design

We used a 3 × 3 mixed design. The between-subjects factor was the number of rehearsed exemplars (out of six) in the retrieval practice phase (with three levels: one, three, or five exemplars). The within-subject factor was the retrieval practice status of each item (with three levels: Rp+, Rp−, and Nrp). The dependent variable was the proportion of items recalled in the final memory test.

#### Material and apparatus

The material and apparatus were identical to those used in Experiment 3A.

#### Procedure

The procedure was identical to that described in the three repetitions group of Experiment 3A except that the number of exemplars cued within each practiced category in the retrieval practice phase was either one, three, or five exemplars (out of 6) in the one-exemplar group, three-exemplar group, or five-exemplar group, respectively. In all three groups, each retrieval practice cue was shown three times, in different random orders. It should also be noted that the duration of the filler task was varied (with one, three, and five exemplars the total time was 16, 12, and 8 min, respectively) to equate the duration from the end of the study phase to the beginning of the final memory test phase across the three groups.

### Results and Discussion

Table 1 shows the mean proportion of exemplars recalled in the final category-cued recall task of Experiment 3B. The data are reported by the retrieval practice status of the exemplar, and whether one, three, or five exemplars from a practiced category had each been cued in the retrieval practice phase. A 3 × 3 mixed ANOVA was performed where the between-subjects factor was the number of exemplars that were cued in a practiced category (one-exemplar group, three-exemplar group, and five-exemplar group), and the within-subject factor was the retrieval practice status of the items with three levels (Rp+, Nrp, and Rp−).

There was a significant main effect of retrieval practice status, *F*(2, 234) = 238.5, *MSE* = .021, η_*p*_^2^ = .671, *p* < .001; a significant main effect of the number of exemplars, *F*(2, 117) = 3.47, *MSE* = .052, η_*p*_^2^ = .056, *p* = .034; and a significant interaction between the two factors, *F*(4, 234) = 4.845, *MSE* = .021, η_*p*_^2^ = .076, *p* = .001.

In examining the interaction, we first examined the effects of retrieval practice and RIF separately for each of the three different groups. Considering first recall in the one-exemplar group, paired *t* tests showed that Rp+ items were recalled significantly more often than Nrp items, *t*(39) = 12.22, *p* < .001, but Rp− items were not recalled significantly less often than Nrp items, *t*(39) = 1.68, *p* = .102, showing significant retrieval practice but nonsignificant RIF effects. Considering next recall in the three-exemplar group, paired *t* tests showed that Rp+ items were recalled significantly more often than Nrp items, *t*(39) = 12.22, *p* < .001, and Rp− items were recalled significantly less often than Nrp items, *t*(39) = 2.15, *p* = .038, showing significant retrieval practice and significant RIF effects. Finally, in the five-exemplar group, paired *t* tests showed that Rp+ items were recalled significantly more often than Nrp items, *t*(39) = 10.30, *p* < .001, and Rp− items were recalled significantly less often than Nrp items, *t*(39) = 5.03, *p* < .001, showing significant retrieval practice and significant RIF effects.

To further explore the interaction, we then calculated for each group the benefit in recall through retrieval practice by subtracting the Nrp values from the Rp+ values. A between-subjects ANOVA examining the magnitude of the recall benefits across the three groups revealed that there was a significant effect of number of practiced exemplars, *F*(2, 117) = 10.68, *MSE* = .026, η_*p*_^2^ = .154, *p* < .001. Independent samples *t* tests revealed that there was greater benefit to recall of retrieval practice in the one-exemplar group (+.374) than in the five-exemplar group (+.208), *t*(78) = 4.53, *p* < .001, and there was greater benefit to recall of retrieval practice in the three-exemplar group (+.299) than in the five-exemplar group (+.208), *t*(78) = 2.87, *p* = .005. However, a further independent samples *t* test showed that there was no significant difference in the benefit to recall due to retrieval practice in the one-exemplar group (+.374) than in the three-exemplar group (+.299), *t*(78) = 1.92, *p* = .059.

We similarly calculated for each group the reduction in recall through RIF by subtracting the Rp− values from the Nrp values. A between-subjects ANOVA examining the magnitude of the reduction in recall across the three groups revealed that there was a significant effect of number of practiced exemplars, *F*(2, 117) = 7.55, *MSE* = .041, η_*p*_^2^ = .114, *p* = .001. Independent samples *t* tests revealed that there was greater reduction in recall due to RIF in the 5-exemplar group (.202) than either the 1-exemplar group (+.040), *t*(78) = 3.48, *p* = .001, or the 3-exemplar group (.064) *t*(78) = 2.78, *p* = .007. However, a further independent samples *t* test showed that there was no difference in the reduction due to RIF in the 1-exemplar group (.040) than in the 3-exemplar group (.064), *t*(78) = 0.63, *p* = .533.

Overall, these data show that the effects of retrieval practice were present no matter how many exemplars per category were practiced, but the magnitude of the effect increased when fewer exemplars per category were practiced. By contrast, the effects of RIF were significant only when three or five exemplars per category were practiced, and the level of RIF increased when 5 related exemplars per category were practiced.

Relating our findings to an end-of-day review, our data suggest that if one wants to maximize the likelihood of remembering a specific event, one should practice that item together with as few as possible of the other related items. By contrast, if one wants to maximize the forgetting of a specific event, then our data suggest that the most effective way of reducing its recall is to practice as many as possible of the other related items to the one to be forgotten.

## Experiment 3C

In Experiment 3B, we found that increasing the number of exemplars of each category that were practiced during retrieval practice decreased the advantage due to retrieval practice but increased the degree of RIF. However, it is unclear whether these effects depended on the absolute number of items that were practiced irrespective of the total number of exemplars in the category, or whether the magnitude of the effects depended on the proportion of practiced items from a category that underwent retrieval practice. Because different events in the real world are likely to have a greater or lesser number of related event exemplars, it would be important to know how different schedules might need to vary when the category set size varies.

In Experiment 3C, we manipulated the total number of studied exemplars in each category, while keeping constant the number of rehearsed exemplars. Specifically, half of the participants studied eight categories of six exemplars each in the study phase of the experiment, and then they underwent retrieval practice of half of the exemplars (three out of six) from half of categories. By contrast, the remaining participants studied 12 exemplars for each of the eight categories, and then rehearsed one quarter (three out of 12) of the exemplars of half of the categories. In this design, the absolute number of cued items in retrieval practice remained the same in the two conditions, while the proportion of the studied items that were practiced was varied.

### Method

#### Participants

Eighty students from the University of Essex were paid £5 in return for participation. The experiment was approved by the research ethics committee of the University of Essex.

#### Design

The experimental design consisted of a 2 × 3 mixed design. The between-subjects factor was the category set size (with two levels: six-exemplar group and 12-exemplar group). The within-subjects variable was the retrieval practice status of each item (with three levels: Rp+, Rp−, and Nrp). The dependent variable was the proportion of items recalled in the final memory test.

#### Materials and apparatus

For each category, the stimulus set was increased to 12 exemplars per category, and the complete stimulus set can be found in Appendix C. Participants in the 12-exemplar group condition saw all 12 exemplars of each category. Participants in the six-exemplar group condition saw six exemplars that were randomly selected from the 12 of each category. The apparatus was identical to that used in Experiment 3A.

#### Procedure

The procedure in the six-exemplar group was identical to that used in the three repetitions group of Experiment 3A. The only difference was that participants in the 12-exemplar group saw all 12 exemplars of each category at study.

### Results and Discussion

Table 1 shows the mean proportions of exemplars recalled in the final category-cued recall task. The data are separated by the retrieval practice status of the exemplar, and whether participants studied 6 or 12 exemplars per category.

A 3 (retrieval practice status) × 2 (group: six exemplars; 12 exemplars) mixed ANOVA showed there was a significant main effect of retrieval practice status, *F*(2, 156) = 173.6, *MSE* = .017, η_p_^2^ = .690, *p* < .001; a significant main effect of category size, *F*(1, 78) = 12.6, *MSE* = .051, η_p_^2^ = .140, *p* = .001; and a nonsignificant interaction, *F*(2, 156) = .914, *MSE* = .017, η_p_^2^ = .012, *p* = .403. Paired *t* tests on the main effect of retrieval practice status revealed a positive effect of retrieval practice on recall (Rp+ was significantly greater than Nrp, *t*(79) = 14.0, *p* < .001), as well as an overall significant effect of RIF (Rp− items were significantly less recalled than Nrp ones, *t*(79) = 2.98, *p* = .004).

For completeness, we additionally examined separate tests of retrieval practice and RIF for each condition, even though the interaction was not significant. Considering first recall in the six-exemplar category group, paired *t* tests showed that Rp+ items were recalled significantly more often than Nrp items, *t*(39) = 9.96, *p* < .001, but Rp− items were not recalled significantly less often than Nrp items, *t*(39) = 1.10, *p* = .279, showing significant retrieval practice but nonsignificant RIF effects.

Considering next recall in the 12-exemplar category group, paired *t* tests showed that Rp+ items were recalled significantly more often than Nrp items, *t*(39) = 10.05, *p* < .001, and Rp− items were recalled significantly less often than Nrp items, *t*(39) = 4.45 *p* < .001, showing significant retrieval practice and significant RIF effects. Furthermore, we then calculated for each group the benefit in recall through retrieval practice by subtracting the Nrp values from the Rp+ values. An independent samples *t* tests revealed that there was no significant difference in the benefit to recall of retrieval practice in the six-exemplar category group (+.329) compared with in the 12-exemplar category group (+.275), *t*(78) = 1.28, *p* = .206. A further independent samples *t* test showed that there was no significant difference in the reduction in recall due to RIF in the six-exemplar category group (.031) than in the 12-exemplar category group (.064), *t*(78) = 1.02, *p* = .312.

Overall, the data from Experiment 3C suggests that both the magnitude of the facilitation caused by retrieval practice and the magnitude of the attenuation in recall caused by RIF are broadly unaffected by the size of the category set. The effect of retrieval practice was substantial and present to a comparable degree in both the six-exemplar and the 12-exemplar categories. The effect of RIF was relatively weak and occurred to a comparable degree in both the six-exemplar and the 12-exemplar categories. However, considering the individual groups separately, the effect of RIF reached significance in the 12-exemplar category group but not in the six-exemplar category group. Thus, there is no evidence that increasing the number of exemplars reduced the magnitude of the retrieval practice or RIF effect; if anything the evidence suggests that the effect was, if anything, marginally (although not significantly) greater when there was a greater number of exemplars.

## Experiment 4

The findings from the laboratory-based Experiments 3A to 3C provided promising evidence that manipulations exist that could be used to maximize and minimize the recall of specific episodes in an end-of-day review. In order to maximize the possibility of later recalling an item, one might seek to practice only that specific item (Experiment 3B) on multiple occasions (Experiment 3A). To minimize the possibility of later recalling an item, one might seek to practice as many other items as possible that are related to that specific item (Experiment 3B), although the number of repetitions of this set of items might not be so important (Experiment 3A). In both cases, the magnitude of these effects might be expected to be relatively unaffected by the total number of related items (Experiment 3C).

The main purpose of Experiment 4 was to examine whether it was possible to apply these laboratory findings to more real-world environments to influence the degree of retrieval practice and RIF through the manipulation of the retrieval practice schedules. We returned to the campus scavenger hunt method that we had used in Experiment 2, in which participants were presented with eight different experimental category names (e.g., “something orange,” “something with stripes”) and had to take a digital image and record an associated comment about each of six different exemplars that they found on campus.

To see whether we could maximally enhance the recall of specific items, one half of the participants received a maximize remembering schedule intended to maximize recall of a specific subset of items (Rp+). Specifically, a single item (selected at random from the set of six) from a random half of the categories received six repetitions of retrieval practice. This was expected to maximize the likelihood of recalling the specific item from each category that received retrieval practice. To see whether we could maximally attenuate the recall of specific items, the remaining participants received a maximize forgetting schedule, in which five items (selected at random from the set of six) from half of the categories received three repetitions of retrieval practice. This was expected to maximize the likelihood of forgetting the one remaining item from each practiced category that had not undergone retrieval practice.

### Method

#### Participants

A total number of 80 students from the University of Essex, participated in this experiment in exchange for either course credit or £15. The experiment was approved by the research ethics committee of the University of Essex.

#### Design

The experiment used was a 2 × 3 mixed factorial design. The between-subjects independent variable was the retrieval practice schedule (with two levels: maximize remembering schedule and maximize forgetting schedule). The within-subject independent variable was the retrieval practice status of each item (with three levels: Rp+, Rp−, and Nrp). The dependent variable was the proportion of correctly recalled items from each of the categories.

#### Materials, apparatus, and procedure

The materials and apparatus were identical to those used in Experiment 2. The procedures for the study, filler and test phases were almost identical to those used in Experiment 2. The only difference was that in the retrieval practice phase, the participants in the maximize remembering schedule were cued with only one exemplar for each of four experimental categories on six occasions, whereas the participants in the maximize forgetting schedule were cued with five exemplars for each of four experimental categories for three occasions. In both retrieval practice schedules, the cues consisted of the experimenter category and the digital images of specific exemplars that had been taken by the participant and shown for 10 s, and participants had to name the object and retrieve aloud the associated comment that they had recorded for the specific item.

Finally, because the total number of cues presented in the retrieval practice phase was different in the two retrieval practice conditions, the duration of the filler task following retrieval practice was adjusted so that the retention interval between the start of retrieval practice and the beginning of the final memory test was the same in the two conditions. At test, participants were presented with each category name in a random order and were given 30 s to recall as many of the to-be-remembered objects from that category as possible.

### Results

The mean proportions of items recalled in the final recall test are shown in Table 1. A 2 × 3 mixed ANOVA revealed that the main effect of retrieval practice schedule was not significant, *F*(1, 78) = 2.60, *MSE* = .049, η_p_^2^ = .032, *p* = .110; the main effect of retrieval practice status was significant, *F*(2, 156) = 39.5, *MSE* = .024, η_p_^2^ = .336, *p* < .001; and there was a significant interaction between the two factors, *F*(2, 156) = 4.43, *MSE* = .024, η_p_^2^ = .054, *p* = .013.

To analyze the significant interaction between the two factors, we compared the mean scores of Nrp items with Rp+ and Rp− items, respectively, with separate analyses for each conditions (paired *t* tests). In the maximize remembering schedule, the difference between Nrp (.644) and Rp+ (.850) items was significant, *t*(39) = 5.77, *p* < .001, showing retrieval practice effects, while the difference between Nrp and Rp− (.643) items was not significant, *t*(39) = .054, *p* = .957. In the maximize forgetting schedule, the difference between Nrp (.681) and Rp+ (.766) items was also significant, *t*(39) = 4.98, *p* < .001, and so was the difference between Nrp and Rp− (.550) items, *t*(39) = 3.45, *p* = .001.

To determine whether the positive effects of retrieval practice on the Rp+ items were greater with the maximize remembering schedule, we calculated the benefit due to retrieval practice for all participants by subtracting the proportion of Nrp items they recalled from their proportion of Rp+ items recalled. The mean benefit of retrieval practice for those participants who viewed the maximize remembering schedule (.206) was significantly greater than the mean benefit of retrieval practice for those participants who viewed the maximize forgetting schedule (.085), *t*(78) = 3.06, *p* = .003. Similarly, to determine whether there was greater RIF in the maximize forgetting schedule, we subtracted the proportion of Rp− items recalled by each participant from their corresponding proportion of Nrp items recalled. The mean loss due to RIF for the maximize forgetting schedule (.131) was significantly greater than the mean loss due to RIF for the maximize remembering schedule (.001), *t*(78) = 2.84, *p* = .006.

### Discussion

Experiment 4 showed that the patterns of retrieval practice and RIF effects observed using real world stimuli are highly consistent with the effects observed in the laboratory in Experiment 3. Most pertinently, in the maximize remembering schedule (where one exemplar of a category was practiced six times) there was significant retrieval practice but the RIF effect was not significant; findings comparable to the one practiced exemplar group in Experiment 3B. Similarly, in the maximize forgetting schedule (where five exemplars of a category were each practiced three times) there was a significant RIF effect and an attenuated effect of retrieval practice; findings comparable to the five practiced exemplar group in Experiment 3B.

Thus, Experiment 4 showed that through changes in the schedules of retrieval practice, we could moderate the magnitude of retrieval practice and RIF effects. Specifically, using a maximize remembering schedule, we could enhance the recall of a specific item through repeated and specific targeted retrieval practice of that item, and using a maximize forgetting schedule, we could attenuate the recall of a specific item through the retrieval practice of as many related items as possible.

It should be noted that although participants selected the stimuli in Experiments 2 and 4, they did not have any control over which items received the different schedules of retrieval practice. Although an advanced, interactive review system might come to know user preferences and so might automatically select the items to be practiced, it is highly likely that it would be beneficial for participants to exert some control over which items they wished to remember and which items they wished to forget. This participant-based control was introduced in Experiment 5.

## Experiment 5

In Experiment 5, we sought to determine whether participants could control the later accessibility of experienced items. Previously, the exemplars targeted for retrieval practice and RIF were selected at random by the experimenter. In Experiment 5, participants were asked to capture nine stimuli from nine experimental categories on a campus scavenger hunt. At the moment of capture, participants were requested to record an additional audio comment about the object.

Following the capture of all the stimuli from all the categories, participants were represented with all nine images in a 3 × 3 grid from each category in turn. Next to each of the nine images was a pair of check boxes (labeled R and F), and participants selected three items from each category that they wished to remember (by checking three R boxes) and three items from each category that they wished to forget (by clicking three F boxes). The three remaining items that were unselected were, by default, considered to be neutral items.

We anticipated that the participants’ selection and intention to remember and to forget study items could in and of itself have later mnemonic effects on the later accessibility of the items. First, participants could select to remember images that were somehow their favorite and more memorable items, irrespective of any differences in subsequent retrieval practice schedules. For example, participants might select to remember those items with the highest image quality, those that were the most artistic or creative, those which the participants were most proud of the inventiveness of assigning the item to the category, or simply those that were captured first or last within each category. Similarly, participants could select to forget those images that were somehow their least favorite and least memorable items, that were poor quality or considered unimaginative, uncreative or from middle serial positions.

Second, the act of assigning the remembering and forgetting labels might result in changes in memorability, irrespective of later retrieval schedules. It is known from studies using the directed forgetting paradigm that individual items selected by the experimenter that participants are told to remember will be recalled better than those participants are told to forget (e.g., Basden & Basden, 1998; MacLeod, 1999). In our experiment, those represented images that participants selected to be remembered might be reencoded differently at selection to those labeled to be forgotten, and participants might choose to spontaneously rehearse or retrieve to-be-remembered items more than to-be-forgotten items.

To determine the separate effects of different retrieval practice schedules and the intention to remember, and to examine their interaction, participants in Experiment 5 were asked to hunt for 9 exemplar objects for 9 experimental categories, and then following stimulus collection, they were asked to review the 9 images captured within each category. During the review of each category, the participants were presented with their captured photos in a 3 × 3 array, and next to each image there were two check boxes marked R and F. Participants were required to select exactly three exemplars to remember, three exemplars to forget, and leave unchecked three (neutral) exemplars from the set of 9 objects photographed by the participant from all the experimenter categories. For each participant, three of the nine categories (selected at random) would receive a maximize remembering retrieval practice schedule, a further random set of three of the nine categories would in addition receive a maximize forgetting retrieval practice schedule, and the remaining three of the nine categories received no retrieval practice.

All participants performed retrieval practice on 108 images. For each image, participants saw the name of an experimental category, an image, and a request to recall the name of the objects and its associated comment. The set of 108 practiced images contained 54 images associated with the maximize remembering schedule and 54 images associated with the maximize forgetting schedule.

Three categories were randomly assigned to the maximize remembering retrieval practice schedule, and participants were presented with the three to-be-remembered exemplars from each of these three categories. Each of the 9 images associated with the maximize remembering schedule received six opportunities for retrieval practice (54 practiced items). A further three categories were randomly assigned to the maximize forgetting retrieval practice schedule, and participants were presented with retrieval practice opportunities for each of the six exemplars that were not marked as to-be-forgotten (i.e., the three to-be-remembered items and the three neutral exemplars) from each of the three randomly selected categories. Each of these 18 images associated with the maximize forgetting schedule was practiced three times (54 practiced items). Finally, the three remaining categories were assigned to the no retrieval practice schedule: None of these exemplars received any retrieval practice opportunities.

Using this method, we could determine the effects caused by deciding to remember and forget items and see how these selections interacted with different retrieval practice schedules aimed at facilitating and attenuating later accessibility. Finally, half of the participants (in the one session of retrieval practice [1RP] group) received a single session of retrieval practice approximately 3 hr after the end of the study phase. The remainder (in the two session of retrieval practice [2RP] group) received two sessions of retrieval practice: one commenced about 1.5 hr after the end of the study phase, and the second session commenced approximately 3 hr after the end of the study phase. Participants in the 2RP group were free to leave the laboratory during the interval between the two retrieval practice sessions.

### Method

#### Participants

A total number of 80 students from the University of Essex, participated in this experiment in exchange for either course credit or £25. The experiment was approved by the research ethics committee of the University of Essex.

#### Design

The study used a 2 × 3 × 3 mixed design. The between-subjects variable was the number of retrieval practice sessions completed by the participants (with two levels: 1RP and 2RP). There were two within-subjects independent variables: participants’ memory preference (with three levels: remember items that were selected to-be-remembered, unselected neutral items, and forget items that were selected to-be-forgotten), and retrieval practice schedule (with three levels: maximize remembering schedule, maximize forgetting schedule, and no retrieval practice schedule).

#### Stimuli and apparatus

There were nine experimental categories and no filler categories. The experimental categories were “something white,” “something made of plastic,” “something rectangular,” “something with numbers,” “something smaller than your hand,” “something shiny,” “something suitable for vegetarians,” “something that moves,” and “something that makes a noise”. Participants were equipped with a Samsung Galaxy SIII mini and used the My Good Old Kodak application (Niforatos et al., 2014) to take photographs, which was downloaded from the Google Play Store. All the remaining tasks were performed using the SuperCard 4.7 application and responses for the recall phase were written down on the provided response sheets (one sheet for each category).

#### Procedure

All participants took part in two sessions, with the first session taking place in the morning, and the second session in the afternoon of the same day. The participants first reported to the experimenter in the laboratory where they were all equipped with a smartphone, shown how to use the My Good Old Kodak camera application and provided with a randomly ordered list of categories. They were initially instructed to search around campus for nine exemplars of each of the nine categories. For each item, they were to take a photograph and record a comment about that object, which was recorded with the Voice Recorder application on the mobile phone. They were instructed to search for objects in the same order as on the experimenter’s list, and they were asked to complete each category before moving on to the next. The My Good Old Kodak application was set up to provide nine rolls of camera film with nine shots on each film. Participants were given a maximum of 150 min to complete this task. Upon their return, the participants completed a short filler task for 10–15 min while the experimenter unlocked and then transferred the images from the smartphones to the computer. Then the participants were shown successive displays of all nine photos of each category, arranged in a 3 × 3 matrix. Adjacent to each image there were two small checkboxes, one labeled “Forget” and one labeled “Remember”, and participants were asked to view the nine images and select for each category (by clicking on those boxes), three photos to forget and three photos to remember. Once the participant had completed the task for all nine categories they could leave the laboratory and then came back later in the afternoon for the second session.

The second session included either one or two sessions of retrieval practice, a filler task and a final memory test. In the 1RP condition, a single session of retrieval practice was performed about three hours after the end of the first study session. In the 2RP condition, two retrieval practice sessions were performed: the first, about 1.5 hr after the study session; and the second, about three hours after the end of the first session. Participants in the 2RP condition were free to leave the laboratory in the interval between the retrieval practice sessions.

During retrieval practice, the computer randomly assigned three of the nine categories to the maximize remembering retrieval practice schedule, three further categories to the maximize forgetting retrieval practice schedule, and the remaining three categories to the no retrieval practice schedule. In each retrieval practice session, participants were presented with a total of 108 retrieval practice opportunities, in which an image was presented for 10 s, together with the name of the experimental category, and the participants were required to try to recall the name of the object and the associated comment that was recorded at the time of the item’s encoding. The 108 presented images consisted of 54 images associated with the maximize remembering retrieval practice schedule and 54 images associated with the maximize forgetting retrieval practice schedule. For the maximize remembering schedule, the three remember exemplars selected by the participants to-be-remembered from each of the three randomly selected categories were practiced six times. For the maximize forgetting schedule, the three remember and the three neutral exemplars from each of three randomly selected categories were each practiced three times.

For those participants in the 2RP condition, the set of presented stimuli and randomization of the second session of retrieval practice was identical to the first session of retrieval practice. All participants then performed a filler task and the final category-cued-recall test that was identical to that used in Experiment 2 and 4. Thus, at test, participants were presented with each of the category names in random order and they were given 30 s to recall as many of the photographed objects from that category as they could remember in any order that they liked.

### Results

Table 2 summarizes the main findings of the test phase of Experiment 5. A 2 × 3 × 3 mixed ANOVA was performed on the recall data, with number of retrieval practice sessions as a between-subjects factor (with two levels: 1RP or 2RP), and two within-subjects independent variables: participants’ memory preference (with three levels: remember, neutral, and forget), and retrieval practice schedule (with three levels: maximize remembering schedule, maximize forgetting schedule, and no retrieval practice schedule). This revealed a nonsignificant main effect of number of retrieval practice sessions, *F*(1, 78) = .037, *MSE* = .140, η_p_^2^ < .001, *p* = .848, a nonsignificant main effect of retrieval practice schedule, *F*(2, 156) = 1.38, *MSE* = .030, η_p_^2^ = .017, *p* = .255, and a significant main effect of participants’ memory preference, *F*(2, 156) = 124.3, *MSE* = .036, η_p_^2^ = .614, *p* < .001.[Table-anchor tbl2]

The 2-way interaction between number of retrieval practice sessions and retrieval practice schedule was nonsignificant, *F*(2, 156) = .493, *MSE* = .030, η_p_^2^ = .006, *p* = .612, the two-way interaction between number of retrieval practice sessions and participants’ memory preference was nonsignificant, *F*(2, 156) = 1.11, *MSE* = .036, η_p_^2^ = .014, *p* = .333, but the interaction between participants’ memory preference and retrieval practice schedule was significant, *F*(4, 312) = 26.6, *MSE* = .027, η_p_^2^ = .255, *p* < .001. The three-way interaction was not significant, *F*(4, 312) = .394, *MSE* = .027, η_p_^2^ = .005, *p* = .813. Because there was no significant main effect or interaction involving the number of sessions of retrieval practice, this variable was removed from the follow up analyses.

Two sets of analyses examined the critical interaction between participants’ memory preference and retrieval practice schedule: a first set considered the effects of participants’ selected memory preferences on recall for each of the three retrieval practice schedules, and a second set compared the effects of the different retrieval practice schedules on recall for each of the participants’ selected memory preferences.

Considering first the analyses of the effects of participants’ selected memory preferences for those categories that received no retrieval practice, a one-way ANOVA revealed a significant effect of memory preference, *F*(2, 158) = 16.38, *MSE* = .027, η_p_^2^ = .172, *p* < .001. Paired *t* tests revealed that the recall of the Remember items that were selected to-be-remembered (.604) was significantly greater than the Forget items that were selected to be forgotten (.471), *t*(79) = 5.27, *p* < .001, and was also significantly greater than those remaining, unselected, neutral items (.479), *t*(79) = 4.43, *p* < .001. A further paired *t* test revealed that there was a nonsignificant difference in recall between the forget items and the neutral items, *t*(79) = 0.34, *p* = .738. These analyses show that there is an improvement in recall for items that are labeled to-be-remembered, even in the absence of any further intervention attributable to a retrieval practice schedule. By contrast, there was no analogous decrement in recall of the items labeled to-be-forgotten in the no retrieval practice condition.

We consider next the effects of participants’ selected memory preferences for those categories that received the maximize remembering retrieval practice schedule. A one-way ANOVA revealed a significant effect of memory preference, *F*(2, 158) = 100.1, *MSE* = .033, η_p_^2^ = .559, *p* < .001. Recall that under this retrieval practice schedule, the Remember items that were selected to-be-remembered each received six opportunities for retrieval practice. Paired *t* tests revealed that the recall of the remember items (.771) was significantly greater than the forget items that were selected to-be-forgotten (.394), *t*(79) = 12.31, *p* < .001. Recall of the remember items was also significantly greater than those remaining neutral items (.446), *t*(79) = 11.43, *p* < .001. A further paired *t* test revealed that there was a nonsignificant decrease in recall between the forget items and those remaining neutral items, *t*(79) = 1.87, *p* = .065. It appears that the maximize remembering retrieval practice schedule greatly increased the recall of the to-be-remembered items relative to the neutral items and resulted in a small but nonsignificant decrease in the recall of the to-be-forgotten items.

We conclude our first set of analyses by considering the effects of participants’ selected memory preferences on those categories that received the maximize forgetting retrieval practice schedule. A one-way ANOVA revealed a significant effect of memory preference, *F*(2, 158) = 73.18, *MSE* = .029, η_p_^2^ = .481, *p* < .001. Paired *t* tests revealed that the recall of the remember items that were selected to-be-remembered (.659) was significantly greater than the forget items that were selected to-be-forgotten (.357), *t*(79) = 12.31, *p* < .001, and also significantly greater than those remaining neutral items (.614), *t*(79) = 2.00, *p* = .049. A further paired *t* test revealed that there was a significant decrease in recall between the forget items and those unselected neutral items, *t*(79) = 8.56, *p* < .001. It appears that the maximize forgetting retrieval practice schedule greatly decreased the recall of the to-be-forgotten items relative to the neutral items but resulted in a small and significant increase in the recall of the to-be-remembered items.

We now consider the second set of analyses examining the effect of the retrieval practice schedules on items that received different participant-selected memory preferences. We start with an analysis of the effect of retrieval practice schedule on the remember items, those items selected by participants to-be-remembered. The remember items received six retrieval practice opportunities in the maximize remembering retrieval practice schedule, three retrieval practice opportunities in the maximize forgetting retrieval practice schedule, and no retrieval practice opportunities in the no retrieval practice schedule. A one-way ANOVA revealed a significant effect of retrieval practice schedule, *F*(2, 158) = 23.02, *MSE* = .025, η_p_^2^ = .226, *p* < .001. Paired *t* tests revealed that the recall of the remember items that received the maximize R retrieval practice schedule (.771) was significantly greater than the recall of those remember items that had received the maximize forgetting retrieval practice schedule (.659), *t*(79) = 4.60, *p* < .001, and also significantly greater than those remember items that received no retrieval practice (.604), *t*(79) = 6.91, *p* < .001. A further paired *t* test revealed that there was a significant difference between recall of the remember items that received the maximize forgetting retrieval practice and the recall of the remember items that received the no retrieval practice, *t*(79) = 2.07, *p* = .042. These analyses reveal that the recall of the to-be-remembered items (which were already high, based on the participants’ remember selection) are further improved by increasing degrees of retrieval practice, with significant increases in recall following six (maximize remembering) and three (maximize forgetting) retrieval practice opportunities over none (no retrieval practice).

We continue our second set of analyses by examining the effect of retrieval practice schedules on the neutral items, those items that were neither selected by participants to-be-remembered nor selected to-be-forgotten. The neutral items do not receive retrieval practice opportunities in the maximize remembering retrieval practice schedule or the no retrieval practice schedule, but they do receive retrieval practice in the maximize forgetting retrieval practice schedule. A one-way ANOVA revealed a significant effect of retrieval practice schedules, *F*(2, 158) = 19.43, *MSE* = .033, η_p_^2^ = .197, *p* < .001. Paired *t* tests revealed that the recall of the neutral items that received the maximize forgetting retrieval practice schedule (.614) was significantly greater than those that received the maximize remembering retrieval practice schedule (.446), *t*(79) = 5.53, *p* < .001, and also significantly greater than those that received no retrieval practice (.479), *t*(79) = 4.60, *p* < .001. A further paired *t* test revealed that there was a nonsignificant difference between recall of the neutral items receiving no retrieval practice and the recall of the neutral items receiving the maximize remembering retrieval practice schedule, *t*(79) = 1.30, *p* = .199. These analyses revealed a significant increase in the recall of the neutral items following the three opportunities each for retrieval practice in the maximize forgetting retrieval practice schedule relative to the maximize remembering retrieval practice schedule and the no retrieval practice schedule, where there was no retrieval practice of the neutral items.

We conclude the second set of analyses by examining the effect of the retrieval practice schedules on the forget items, those items selected by participants as to-be-forgotten. The forget items receive no retrieval practice opportunities in any of the retrieval practice schedules, but might be expected to undergo greater forgetting in the maximize forgetting retrieval practice schedule where 6 different same-category exemplars were practiced three times each, relative to the maximize remembering retrieval practice schedule where 3 different same-category exemplars were practiced six times each, relative to the no retrieval practice schedule where no same-category exemplars were practiced. A one-way ANOVA revealed a significant effect of retrieval practice schedules, *F*(2, 158) = 10.43, *MSE* = .026, η_p_^2^ = .117, *p* < .001. Paired *t* tests revealed that the recall of the Forget items that received the no retrieval practice schedule (.471) was significantly higher than those that received the maximize forgetting retrieval practice schedule (.357), *t*(79) = 4.37, *p* < .001, and also significantly higher than those that received the maximize remembering retrieval practice schedule (.394), *t*(79) = 3.05, *p* = .003. A further paired *t* test revealed that there was a nonsignificant difference between recall of the forget items receiving the maximize forgetting retrieval practice schedule and the recall of the forget items receiving the maximize remembering retrieval practice schedule, *t*(79) = 1.49, *p* = .140. These analyses suggest that to-be-forgotten items can exhibit retrieval-induced forgetting, and that the magnitude of the forgetting is almost equivalent whether there are 18 same-category retrieval practice opportunities spread across six different exemplars (maximize forgetting retrieval practice schedule) or there are 18 same-category retrieval practice opportunities concentrated on three different exemplars (maximize remembering retrieval practice schedule).

We conducted a final pair of comparisons to examine the combined effectiveness of both the participants’ preference and the retrieval practice schedule on subsequent recall. As the bold values in Table 2 show, the greatest recall was for remember items that received the maximize remembering retrieval practice schedule (.771), and this was significantly higher than the baseline recall values of neutral items that received no retrieval practice (.479), *t*(79) = 10.2, *p* < .001. By contrast, the lowest recall was for the forget items that received the maximize forgetting retrieval practice schedule (.357), and this was significantly lower than the baseline recall values of neutral items that received no retrieval practice (.479), *t*(79) = 4.48, *p* < .001. Thus, the interactive review was successful at enhancing and attenuating to-be-remembered and to-be-forgotten items, respectively.

### Discussion

Experiment 5 showed that retrieval practice and RIF effects can occur with participant-generated stimuli under conditions in which participants used an interactive review to select which items they wished to remember and which items they wished to forget. In this review, all nine exemplars of each of the nine categories were displayed for the participants, who were asked to select three items to remember and three items to forget (the remaining unselected items were termed the neutral items).

First, our findings showed that those remember items that were selected by participants to-be-remembered were recalled more often than neutral items, even in the absence of any retrieval practice schedule. This could reflect that participants were selecting items to remember that were intrinsically memorable in some way (e.g., due to being an interesting choice of exemplar or due to the perceived quality of the captured image) or could reflect that, once selected, additional postselection processing such as additional rehearsal, reminding, or explicit retrieval of these items. By contrast, there was no memorial effect of indicating a willingness to forget: in the absence of any retrieval practice schedule, participants’ recall was no worse for the forget items that participants selected to-be-forgotten relative to the neutral items. This latter finding was welcome but not entirely guaranteed, since asking participants to forget specific items can be counterproductive (e.g., Wegner, Schneider, Carter, & White, 1987; Wegner, 2011).

Second, we demonstrated that when memory preferences to remember were coupled with the maximize remembering retrieval practice schedule, then significant recall benefits were observed relative to those unselected neutral items. Similarly, when memory preferences to forget were coupled with the maximize forgetting retrieval practice schedule, then significant decrements in recall were observed relative to those unselected neutral items. These findings demonstrate the effectiveness of a prototypical simple interactive review in which participants merely select which items should be remembered or forgotten, and through an automated schedule of retrieval practice, the participant is able to recall spontaneously more or less, respectively in a subsequent test of memory.

Although the interactive review was largely successful, the findings from Experiment 5 were not completely as we had desired. Although the maximize remembering retrieval practice schedule worked well in increasing the recall of the to-be-remembered items to a level above the other two retrieval practice schedules and the maximize forgetting retrieval practice schedule resulted in significant RIF effects for the forget items relative to the forget items in the no retrieval practice schedule, the degree of RIF for these forget items was not significantly greater than the RIF observed on the forget items in the maximize remembering retrieval practice schedule. This contrasts with the findings in Experiment 4, where we had managed to selectively manipulate the remembering and forgetting of single items from a category using the maximize remembering and the maximize forgetting schedules.

Our best explanation for this difference stems from a comparison of the number of items that were attempted to be affected by the retrieval practice schedules and hence the number of potentially RIF-inducing retrieval practice opportunities in the retrieval practice schedules of Experiments 4 and 5. In Experiment 4, there was a threefold difference in the number of retrieval practice opportunities in the two retrieval practice schedules: the maximize remembering retrieval practice schedule received six retrieval practice opportunities of a single exemplar per category (to increase the recall of a single item), whereas the maximize forgetting retrieval practice schedule received three retrieval practice opportunities for each of five exemplars per category (to decrease the recall of a single item). By contrast, the aim of each retrieval practice schedule in Experiment 5 was to try to increase or to decrease the recall of three exemplars. To accomplish this, a total of 54 retrieval practice opportunities were used in both the maximize remembering and the maximize forgetting retrieval practice schedules.

Although we might have expected greater RIF with a greater number of different exemplars (Experiment 3B) rather than greater RIF with a greater number of repetitions of the same exemplars (Experiment 3A), it appears that both the maximize remembering and maximize forgetting schedules in Experiment 5 produced forgetting of approximately equivalent magnitude. One might imagine that had we asked participants to select just one item to remember and one item to forget per category (such that the retrieval practice opportunities across the two retrieval practice schedules were uneven as in Experiment 4), we would have seen selective recall and selective forgetting from the two schedules.

In summary, Experiment 5 showed that it is possible for participants to choose items to-be-remembered and items to-be-forgotten, and that it is possible to fulfil these requirements via the maximize remembering and maximize forgetting retrieval practice schedules. Although more work may be needed to determine the number of exemplars per category that it is possible to enhance or attenuate, selectively, Experiment 5 nevertheless constitutes a successful first attempt at an interactive review designed to facilitate and attenuate the later recall of participant-selected to-be-remembered and to-be-forgotten items.

## Experiment 6

A final experiment, Experiment 6, was performed to check that the retrieval practice effects and retrieval induced forgetting found in Experiments 1 to 5 were not limited to the type of recall test that was used. In all previous experiments, participants were provided at test with a category name (e.g., a campus location or scavenger hunt category) and asked to recall as many studied exemplars of that category as possible, in any order that they liked. This was the type of test used in the original demonstration of RIF (Anderson et al., 1994, Experiment 1) and seemed the most appropriate type of test to use to examine RIF in the real world, because we were primarily interested in whether it was possible to manipulate the spontaneous accessibility to different exemplars when they later thought (in general) about different types of events.

However, it has become more common in studies using the retrieval practice paradigm to follow the example set by Anderson et al. (1994, Experiment 2) and use an extensive set of item-specific cued recall probes at test. In this type of test, participants are tested on each item separately and they are probed with the category name and the first two letters of an exemplar (such as “FRUIT-Or?”).

The method of testing is important when trying to understand the nature of forgetting that occurs following the review of the images. According to the theories of RIF outlined in the introduction, the review of a subset of images in the retrieval-practice phase directly causes a decrease in the accessibilities of individual Rp− items through competition or inhibition. These theories have been formulated following experiments that have shown RIF using both a category cued test of free recall and a series of item-specific cues at test. One advantage of using the item-specific probe methodology is that the accessibility of each item can be assessed separately from other items. Moreover, if the order of the test probes is completely randomized, then any effect of retrieval practice or RIF cannot be attributed to differences in testing order or output interference.

By contrast, when the category-cued free-recall task is used at test, there remains the possibility that the reduction in recall observed on Rp− items is not the direct result of inhibition or competition during the retrieval practice phase, but occurs more indirectly, being driven by test dynamics in free recall. If the practiced items are recalled earlier in tests of free recall, then the reduction in recall of Rp− items may reflect the greater difficulty in attempting to retrieve under conditions of greater output interference (e.g., Roediger, 1973, 1974, 1978). Although both types of mechanism could be considered a type of RIF, it is arguably necessary to demonstrate RIF using both category-cued free recall and item-specific testing methods if we are to have confidence that the theories of RIF raised in the introduction have been applied successfully to the end-of-day review.

Therefore, Experiment 6 was conducted using item-specific probes to clarify the types of mechanisms that are underpinning our findings. If we could observe retrieval practice and RIF using randomized item-specific probes then we would be able to rule out an explanation based solely on output interference and allow us to more confidently argue that theories of RIF outlined in the introduction could be applied in the real world to assist in the augmentation of human memory.

Specifically, we used the same procedure as Experiment 5 but changed the test to an item-specific cued test. Thus, participants were asked to capture nine different items relating to nine different categories. Once participants completed the study phase, their photos were processed prior to naming all 81 captured objects. This was required since the first two letters of each item name were at test used as an item-specific cue. The experimenter therefore ensured that no two first letters within a specific category were used more than once. Once participants had named all the captured items, they were asked to select for each category, three remember items that they wanted to remember items, and three forget items that they wished to forget. We designated the remaining three unselected items from each category as neutral items. In the second session, which occurred in the afternoon of the same day as the study phase, participants received retrieval practice, followed by a filler task, and finally a recall test. Critically, at test, participants were presented with a category name and an item-specific cue (i.e., the first two letters of each item’s name), and their task was to write down the full name of each cued item. All 81 experimental test items were probed one at a time, in a randomized order, using the category name and the first two letters of the exemplar as the cue. Using this method, we were able to eliminate any confounding effects associated with output interference.

### Method

#### Participants

A total number of 40 students from the University of Essex, participated in this experiment in exchange for either course credit or £20. The experiment was approved by the research ethics committee of the University of Essex.

#### Design

The study used a 3 × 3 within-subjects design. There were two within-subjects independent variables: participants’ memory preference (with three levels: remember items that were selected to-be-remembered, unselected neutral items, and forget items that were selected to-be-forgotten), and retrieval practice schedule (with three levels: maximize remembering schedule, maximize forgetting schedule, and no retrieval practice schedule).

#### Materials, apparatus, and procedure

The materials and apparatus were identical to those used in Experiment 5. The procedures for the study phase and the retrieval practice phase were similar to those used in Experiment 5. The only difference in the study phase was that participants upon their return to the laboratory were first asked to name each of the captured photographic images by typing a name into a text box that was present underneath each image. This was performed for all categories, prior to reviewing each category and selecting the three photos to remember and three photos to forget. Participants were required to name the items such that no two items within a single category had identical first two letters. This was because the first two letters of each object were later used as a cue in the item-specific test.

The only difference in the retrieval practice phase was that an item-specific cue was used. Thus, participants saw the photographic image of an item, the category it was associated with, as well as the first two letters of the item’s name. Participants were asked to look at the image, and then say out loud the category name, the item’s name and the associated comment about the item.

Following the filler task (which was identical to that used in Experiment 5), participants completed the test phase. For each studied item, they received an item-specific probe consisting of a category name and the first two letters of the target item. Their task was to write down the full name of each item in the provided booklet. For example, if participants captured a photo of an alarm for the category “Something that makes a noise,” at test they saw “Something that makes a noise—al______,” and their task was to write “alarm” in the provided booklet. All 81 experimental items were tested individually in a randomized order.

### Results

Table 2 summarizes the main findings of the test phase of Experiment 6. A 3 (participants’ memory preference: Remember, Neutral, and Forget) × 3 (retrieval practice schedule: maximize remembering schedule, maximize forgetting schedule, and no retrieval practice schedule) repeated measure ANOVA was performed on the recall data. This revealed a significant main effect of retrieval practice schedule, *F*(2, 78) = 4.34, *MSE* = .035, η_p_^2^ = .100, *p* = .016; a significant main effect of participants’ memory preference, *F*(2, 78) = 47.6, *MSE* = .025, η_p_^2^ = .550, *p* < .001; and a significant interaction, *F*(4, 156) = 13.6, *MSE* = .025, η_p_^2^ = .259, *p* < .001.

We performed two sets of analyses on the significant interaction between participants’ memory preference and the assigned retrieval practice schedule. We first considered the effects of participants’ selected memory preferences on recall for each of the three retrieval practice schedules. A second set of analyses compared the effects of the different retrieval practice schedules on recall for each of the participants’ selected memory preferences.

Considering first the analyses of the effects of participants’ selected memory preferences for those categories that received no retrieval practice, a one-way ANOVA revealed a nonsignificant main effect of memory preference, *F*(2, 78) = 3.05, *MSE* = .026, η_p_^2^ = .073, *p* = .053. This analysis therefore shows that there is no significant improvement in recall for items that are labeled to-be-remembered, and no significant decrement in recall for items labeled to-be-forgotten in the absence of any further intervention attributable to a retrieval practice schedule.

We consider next our analyses on the effects of participants’ selected memory preferences for those categories that received the maximize remembering retrieval practice schedule (i.e., where the remember items each received six opportunities for retrieval practice). A one-way ANOVA revealed a significant effect of memory preference, *F*(2, 78) = 29.9, *MSE* = .024, η_p_^2^ = .434, *p* < .001. Paired *t* tests revealed that the recall of the remember items (.689) was significantly greater than both the forget items (.464), *t*(39) = 6.08, *p* < .001, and the neutral items (.447), *t*(39) = 7.54, *p* < .001. A further paired *t* test revealed that there was a nonsignificant decrease in recall between the forget and the neutral items, *t*(39) = .470, *p* = .641. It appears that the maximize remembering retrieval practice schedule greatly increased the recall of the to-be-remembered items relative to the neutral and the to-be-forgotten items, but the recall of the to-be-forgotten items was not significantly decreased relative to the neutral items.

We conclude our first set of analyses by considering the effects of participants’ selected memory preferences on those categories that received the Maximize Forgetting rehearsal schedule. A one-way ANOVA revealed a significant effect of memory preference, *F*(2, 78) = 42.9, *MSE* = .024, η_p_^2^ = .524, *p* < .001. Paired *t* tests revealed that the recall of the remember items (.653) was significantly greater than the forget items (.367), *t*(39) = 8.00, *p* < .001, but not significantly greater than the neutral items (.639), *t*(39) = .355, *p* = .725. A further paired *t* test revealed that there was a significant decrease in recall between the Forget items and the neutral items, *t*(39) = 9.50, *p* < .001. It appears that the maximize forgetting rehearsal schedule greatly decreased the recall of the to-be-forgotten items relative to the to-be-remembered and the neutral items.

We now consider the second set of analyses examining the effect of the retrieval practice schedules on items that received different participants’ selected memory preferences. We start with an analysis of the effect of retrieval practice on the remember items. The remember items received six specific retrieval practice opportunities in the maximize remembering retrieval practice schedule, and three retrieval practice opportunities in the maximize forgetting retrieval practice schedule and no retrieval practice opportunities in the no retrieval practice schedule. A one-way ANOVA revealed a significant effect of retrieval practice schedules, *F*(2, 78) = 10.6, *MSE* = .032, η_p_^2^ = .213, *p* < .001. Paired *t* tests revealed that the recall of the remember items that received the maximize remembering retrieval practice schedule (.689) was not significantly greater than those that received the maximize forgetting retrieval practice schedule (.653), *t*(39) = 1.15, *p* = .258. However, the recall of the remember items that received the maximize remembering retrieval practice was significantly greater than those that received no retrieval practice (.514), *t*(39) = 4.25, *p* < .001. A further paired *t* test revealed that there was a significant difference between recall of the remember items receiving maximize forgetting retrieval practice and the recall of the remember items that received no retrieval practice, *t*(39) = 2.99, *p* = .005. These analyses reveal that although the recall of the Remember items is improved by retrieval practice opportunities over none (no retrieval practice), the increased number of rehearsals in the maximize remembering schedule did not have a significant improvement on the recall of the to-be-remembered items, relative to the maximize forgetting schedule.

We continue with an analysis of the effect of retrieval practice on the neutral items. The neutral items do not receive retrieval practice opportunities in either the maximize remembering retrieval practice schedule or the no retrieval practice schedule, but they are practiced in the maximize forgetting retrieval practice schedule. A one-way ANOVA revealed a significant effect of retrieval practice schedules, *F*(2, 78) = 14.1, *MSE* = .027, η_p_^2^ = .266, *p* < .001. Paired *t* tests revealed that the recall of the neutral items that received the maximize forgetting retrieval practice schedule (.639) was significantly greater than those that received the maximize remembering retrieval practice schedule (.447), *t*(39) = 5.35, *p* < .001, and also significantly greater than those that received the no retrieval practice schedule (.506), *t*(39) = 3.29, *p* = 002. A further paired *t* test revealed that there was a nonsignificant difference between recall of the neutral items receiving the no retrieval practice schedule and the recall of the neutral items receiving the maximize remembering retrieval practice schedule, *t*(39) = 1.70, *p* = .097. These analyses revealed a significant increase in the recall of the neutral items following the three opportunities each for retrieval practice in the maximize forgetting retrieval practice schedule relative to the maximize remembering retrieval practice schedule and the no retrieval practice schedule, where there was no retrieval practice of the neutral items.

We conclude our second set of analyses by examining the effect of the retrieval practice schedules on the forget items. The forget items received no retrieval practice opportunities in any of the retrieval practice schedules, but might be expected to undergo greater forgetting in the maximize forgetting retrieval practice schedule where six different same-category exemplars were practiced three times each, relative to the maximize remembering retrieval practice schedule where three different same-category exemplars were practiced six times each, relative to the no retrieval practice schedule where no same-category exemplars were practiced. A one-way ANOVA revealed a significant effect of retrieval practice schedules, *F*(2, 78) = 4.03, *MSE* = .025, η_p_^2^ = .094, *p* = .022. A paired *t* test revealed that there was a significant difference between recall of the Forget items that received the maximize forgetting retrieval practice schedule and the recall of the forget items that received the maximize remembering retrieval practice schedule, *t*(39) = 2.80, *p* = .008. However, paired *t* tests revealed that the recall of the Forget items that received the no retrieval practice schedule (.433) was not significantly higher than those that received the maximize forgetting retrieval practice schedule (.367), *t*(39) = 1.98, *p* = .055, and was also not significantly higher than those that received the maximize remembering retrieval practice schedule (.464), *t*(39) = .833, *p* = .410. These analyses suggest that to-be-forgotten items can exhibit retrieval-induced forgetting, and that the magnitude of the forgetting is significantly higher when there are 18 same-category retrieval practice opportunities spread across six different exemplars (maximize forgetting retrieval practice schedule) relative to when there are 18 same-category retrieval practice opportunities concentrated on three different exemplars (maximize remembering retrieval practice schedule).

We conducted a final pair of comparisons to examine the combined effectiveness of both the participants’ preference and the retrieval practice schedule on subsequent recall. As the bold values in Table 2 show, the greatest recall was for remember items that received the maximize remembering retrieval practice schedule (.689), and this was significantly higher than the baseline recall values of neutral items that received no retrieval practice (.506), *t*(39) = 4.79, *p* < .001. By contrast, the lowest recall was for the forget items that received the maximize forgetting retrieval practice schedule (.367), and this was significantly lower than the baseline recall values of neutral items that received no retrieval practice (.506), *t*(39) = 3.45, *p* = .001. Thus, even with item-specific cues, the interactive review was again successful at enhancing and attenuating to-be-remembered and to-be-forgotten items, respectively.

### Discussion

The primary aim of Experiment 6 was to see whether the findings from Experiment 5 could be replicated using a test of recall that used many individual item-specific probes. As outlined in the introduction, the observation of retrieval practice and RIF effects using item-specific probes at test would show that the effects of earlier experiments could not be entirely attributable to differences in output interference and would help clarify that the theories of RIF that were outlined in the introduction may be shown to be extended to the real world.

Overall, Experiment 6 showed that retrieval practice and RIF effects can clearly be produced through the interactive review, even with item-specific cues. Items that participants selected to-be-remembered that received maximize remembering retrieval practice schedules were again the highest recalled (.689, compared with .771 in experiment 5), and the lowest recall was again for items that participants selected to-be-forgotten that received maximize forgetting retrieval practice schedules (.367, compared with .357 in Experiment 5). These values were again significantly higher and significantly lower, respectively, than the baseline recall values of neutral items that received no retrieval practice (.506 compared with .479 in Experiment 5).

The findings of Experiment 6 therefore encourage greater confidence in assuming that the strength of associations between category and exemplars have been directly strengthened and weakened through competition and/or inhibition during the interactive review, and encourage the application of established theories of RIF and laboratory-based findings to the SenseCam and life-logging literatures, and broaden the range of memory cues and retrieval situations in which attenuation and facilitation of experienced events may be expected.

Despite these broad similarities, there were three main differences between the findings of the two experiments, indicating that the change in the testing methodology did indeed affect how participants recalled the different items. First, participants in Experiment 5 who were tested using a category-cued free-recall task recalled a higher proportion of items that they labeled as to-be-remembered, even in the absence of any retrieval practice schedule. By contrast, the participants’ recall of items in Experiment 6 were unaffected by their own memory preferences when tested using item-specific tests of memory.

Second, participants in Experiment 5 who were tested using a category-cued free-recall task recalled nonsignificantly more forget items in the maximize remembering (.394) retrieval practice schedule than in the maximize forgetting (.357) retrieval practice schedules, a finding that had earlier suggested that the retrieval practice schedules did not cause differential rates of forgetting of the Rp− items. By contrast, participants in Experiment 6 who were tested using item-specific probes recalled significantly fewer forget items in the maximize forgetting (.367) retrieval practice schedules than the maximize remembering (.464) schedules.

Finally, participants in Experiment 5 who were tested using a category-cued free-recall task recalled significantly more remember items in the maximize remembering (.771) retrieval practice schedule than in the maximize forgetting (.659) retrieval practice schedules, a finding that had earlier suggested that the retrieval practice schedules caused differential rates of enhancement of the Rp+ items. By contrast, participants in Experiment 6 who were tested using item-specific probes recalled only nonsignificantly more remember items in the maximize remembering (.689) retrieval practice schedule than in the maximize forgetting (.653) retrieval practice schedules.

Together, these differences suggest that the facilitation and attenuation of items due to the retrieval practice phase are somewhat greater when tested using category-cued free recall in Experiment 5 than item-specific cues in Experiment 6. These differences could be attributable, in part, to the additional contribution of output interference in the data from Experiment 5 but not 6. If items strengthened through retrieval practice are also output relatively early on in the category-cued free recall period in Experiment 5, then Rp+ items in Experiment 5 will be attempted to be recalled under conditions of less output interference than the Rp− items, subtly enhancing the recall of the remember items and subtly reducing the recall of the forget items.

It is also possible that the randomized testing order of the probes in Experiment 6 may have disrupted participants’ ability to focus for any length of time on recollecting the episodic study of the categorized items. In Experiment 5, participants were provided with the category cue and underwent a sustained retrieval attempt to free recall exemplars from that category. It may be that this sustained method of testing reminds participants of the choices that they made during the selection of their own memory preferences within each category when they saw all nine items from each category and decided which to remember and which to forget. It may be therefore this sustained retrieval attempt using the category cued free-recall task that encourages the recall of the remember items. By contrast, in Experiment 6 there were no general category cues, and the constant changing between cues to different exemplars from different categories may have encouraged participants to primarily use the item-specific letter cues and their differential retrieval practice schedules and discouraged the use of nonspecific considerations such as the participants’ preferences within each category.

## General Discussion

The overarching aim of these studies was to see whether subsets of data captured, processed, and then displayed through smartphone and lifelogging technologies (e.g., Dodge & Kitchin, 2007; Gurrin et al., 2014; Harvey et al., 2016; Silva et al., 2018) had the potential to be used as an “end-of-day review” (Finley et al., 2011) that could be used to augment human memory by increasing spontaneous accessibility to reviewed items through retrieval practice and by attenuating accessibility to related but nonreviewed items through RIF. To this end, we conducted six experiments and showed that retrieval practice and RIF could indeed occur with more real-world stimuli.

In Experiment 1, we showed significant effects of both retrieval practice and RIF on our experimenter-guided campus tour. Although retrieval practice and RIF effects have been reported in earlier studies with autobiographical memories (e.g., Barnier et al., 2004; Ditta & Storm, 2016; Stone et al., 2013), many of these earlier studies used verbal summaries of events that had taken place prior to the experiment as the to-be-remembered stimuli (e.g., subjectively recollected events that participants had retrieved early in the experiment in response to experimental cues, e.g., recall happy memories). By contrast, Experiment 1 showed retrieval practice and RIF on our experimenter-guided campus tour in which we were able to exert methodological control on the presentation and encoding of all the to-be-remembered events to the participants, and we controlled the selection and presentation of those items to receive retrieval practice.

Experiment 2 showed that retrieval practice and RIF effects could also be observed with participant-generated stimuli in the campus scavenger hunt task. In this method, participants were tasked with taking photographs of, and recording associated comments about, a number of exemplars from different experimenter-generated categories. The photographs served as an objective record of what participants had experienced, and by using the My Good Old Kodak smartphone application, these images could not be reexamined, reviewed or replayed prior to the retrieval practice and test phase.

Experiment 3 showed that we could selectively amplify the effect of retrieval practice through increasing the number of repetitions of retrieval practice (Experiment 3A), and by using highly specific retrieval practice of a single exemplar (Experiment 3B). We also showed that we could selectively amplify the effect of RIF on a target item through increasing the number of different related exemplars that are practiced (Experiment 3B). These manipulations were first observed in the laboratory (Experiment 3) and are consistent with theories of encoding specificity (Tulving, 1983) and cue-overload (Earhard, 1967; Surprenant & Neath, 2009; Watkins & Watkins, 1976), in which the effectiveness of a cue is related to the extent to which it is highly specific and the extent to which it was present at both study and test. We then applied these manipulations to the campus scavenger hunt task (Experimenter 4) such that we could implement one (maximize remembering) schedule of retrieval practice in which we could selectively amplify the degree of retrieval practice for target items and a second (Maximize Forgetting) schedule of retrieval practice in which we could selectively amplify the degree of RIF for those items that were so selected.

In Experiment 5, we were able to implement a prototypical interactive user interface to help augment human memory. For each experimenter-defined category of event, the participants were represented with all nine participant-generated exemplars and they were required to select which of these images they wished to remember and which of these images they wished to forget. Simply selecting an item to remember enhanced its subsequent recall in a category cued free recall test, a finding related to the phenomenon of item-based directed forgetting (e.g., Basden & Basden, 1998). However, recall of the to-be-remembered items that received the maximize remembering retrieval practice schedule was further enhanced, whereas the to-be-forgotten items that received the maximize forgetting (and for that matter, the maximize remembering retrieval practice schedule) were further attenuated through RIF.

In our final experiment, Experiment 6, we attempted to replicate Experiment 5 using a different method of testing. Specifically, we used multiple item-specific probes to test the accessibility of individual items in Experiment 6 rather than test memory using category-cued tests of free recall. The advantage of using item-specific probes is that by testing individual items in a random order, we could rule out any potential confounding effects of output order. We confirmed the presence of retrieval practice and RIF effects using the item-specific probes in Experiment 6, and so can rule out an explanation of our earlier findings that is entirely attributable to output interference. The findings broaden the range of testing conditions over which we might expect to see an effect and provide greater confidence that the types of enhancement and forgetting observed in the real world are similar to those that have to date been explained by the theories of RIF outlined in the introduction.

We believe that these findings are highly promising as an initial attempt at an interactive review schedule to aid in the augmentation of human memory. Our findings further broaden the application of retrieval practice and RIF from the laboratory to the real world, and provide the first applications of the RIF methodology to the lifelogging literature. We believe that retrieval practice and RIF can play an important role in the development of future applications for human memory augmentation systems. Almost certainly, the principle reason for wishing to augment human memory will be to increase recall of the things that we wish to remember. Across the different conditions of our six experiments, the mean mnemonic benefit to later recall was in the order of 25%. The highly reliable finding of retrieval practice effects is consistent with the memorial benefits associated with reviewing SenseCam images in a later test (e.g., Sellen et al., 2007; Silva et al., 2013), and consistent with well-known rehearsal (e.g., Rundus, 1971; Tan & Ward, 2000) and testing effects (Roediger & Karpicke, 2006a; Roediger et al., 2011), more generally, in which later recall benefits from a practiced retrieval to a greater extent than a restudy opportunity. The discovery that represented images can act as a cue to help retrieve associated comments provides some evidence for the recollection-like property of cued recall from images that Silva et al. (2018) were seeking to find.

However, our data also provide a strong indication that retrieval practice based on represented images from a subset of one’s day could, under certain conditions, attenuate later accessibility to related events. By contrast to the retrieval practice effects, this finding has not, as far as we are aware, been previously reported in the SenseCam literature. In many situations, these RIF effects may be unwanted or unexpected. For example, we found that the maximize remembering retrieval practice schedule (designed to enhance later recall of items that were selected as to-be-remembered items) in Experiment 5 resulted in almost the same amount of RIF on the to-be-forgotten items as the maximize forgetting schedule (that was specifically designed to attenuate recall of items selected to-be-forgotten). As discussed, it is worth noting that a potential drawback of trying to enhance selectively the recall of multiple items through retrieval practice is that there may be an undesired reduction in the later accessibility to related but unpracticed items. Our findings from Experiment 4 suggest that one should perform highly specific retrieval practice of single items if one wishes to enhance recall selectively through retrieval practice while minimizing forgetting on related but unpracticed items through RIF. Overall, across the different conditions of our six experiments, the mean mnemonic cost through RIF to later recall was in the order of 8%. Alternatively, the discovery of RIF using a lifelogging style methodology offers the potential for the deliberate development of RIF-inducing retrieval practice schedules to be used during an end-of-day review (Finley et al., 2011) with the explicit aim of attenuating accessibility to the to-be-forgotten items, motivated for personal or potentially even clinical reasons.

Our experiments did not set out to test between competing theories of RIF effects, and indeed, we were far from certain that RIF effects would even be observed. Prior laboratory-based research using semantic category stimuli has shown that the magnitude of the RIF effects is influenced by the strength of the category-exemplar associations (Anderson et al., 1994) and by the coherence of the exemplars (Anderson & McCulloch, 1999; Chan, 2009). By contrast, our real-world category-exemplar stimuli might be argued to have weaker strengths of associations, the different exemplars within a category might be expected to have a greater degree of coherence, and there might be expected to have a greater degree of overlap between exemplars of one category and the exemplars of another category. However, none of these limitations appears to have prevented the observation of RIF with real-world stimuli in our studies.

One important methodological point is that, in all but our last study, the retrieval practice and RIF effects were observed using final tests of recall in which participants were presented with a campus location or an experimenter-defined category (e.g., “something orange”) and participants were free to recall as many target items as they could. It is widely understood that retrieval dynamics can lead to recall being both self-propagating and self-limiting (Roediger, 1973, 1974, 1978). Each of the cued exemplars may be assumed to compete for selection at recall, and recall can progress in a way that is self-propagating (in that recalled items can themselves be used as cues to facilitate the recall of additional items), or self-limiting (if one accepts that recalled items can be resampled, then it may become increasingly difficult to recall an item that has yet to be recalled, a phenomenon known as output interference (e.g., Bäuml, 1998; Laming, 2010; Roediger & Schmidt, 1980; Smith, 1971; Tan & Ward, 2007).

The exact nature of the final test cue is important. If we had given our participants at test a very general retrieval cue, such as to free recall all the items that were observed on campus, then the retrieval practice of a related item from one of the 10 categories or locations might actually facilitate recall of other unpracticed exemplars from that category, because it might remind participants of a whole category or location that they might otherwise have not remembered (cf. Hudson & Austin, 1970). However, given that the items to-be-remembered were all cued with a specific location cue (as in Experiment 1) or a specific category (as in Experiments 2, 4 and 5), then the presentation or retrieval practice of a related item might be expected to attenuate recall of a related item through output interference.

Fortunately, the findings from Experiment 6 strongly suggest that the results of Experiments 1 to 5 cannot be entirely attributable to output interference (e.g., Raaijmakers & Shiffrin, 1980; Rundus, 1973). In Experiment 6, the RIF effects were observed when tested with multiple, item-specific probes, presented in a randomized order. This success with the item-specific cue allows our data to be more easily compared with laboratory studies using this method and makes our data of greater interest to theories of RIF that can account for both methods. Experiment 6 also opens the way for future experiments to discriminate between competing accounts of RIF. It could be possible to determine whether the observed RIF effects were observed even when probed with an independent cue (e.g., Anderson & Spellman, 1995), a finding that if observed would be more readily explained by theories that propose the inhibition of unreviewed but related items.

A second important methodological detail that is present in all our real-world studies (but not our laboratory-based studies) is that participants always studied to-be-remembered items that were paired with associated comments and they always had to try to name the target item and its associated comment during the retrieval practice phase. In an as yet unpublished article (Cinel, Cortis Mack, & Ward, 2018), we show that the requirement to try to retrieve the associated comment during retrieval practice may be a boundary condition in certain situations for observing RIF in laboratory-based studies that used episodic stimuli. Our interpretation is that the requirement to try to retrieve the associated comment necessitates that the participant tries to reinstate the original learning context (e.g., Dando, Wilcock, & Milne, 2009; Sahakyan & Kelley, 2002; Smith & Vela, 2001; Tulving, 1983), and this is critical for the necessary competition and/or inhibition to emerge. It is relatively common in models of episodic memory for multiple items to be associated with the same list context (e.g., Raaijmakers & Shiffrin, 1981; Rundus, 1973; Shiffrin, 1970) or group context (e.g., Farrell, 2012) and our effects of retrieval practice could be interpreted as relating to the competition between to-be-remembered items that are associated to a shared category-specific episodic context (Raaijmakers & Jakab, 2013). The importance of reinstating the relevant study context at retrieval practice and test is fundamental to the Jonker et al. (2013) contextual account of RIF, and, as Anderson (2003) discussed, the extent to which participants cue with contextual information at practice can have large effects on competitive dynamics and hence RIF. Moreover, the neural network account proposed by Norman et al. (2007) proposed a context scale parameter that is necessary to modulate the extent to which participants were actively trying to retrieve memories from a particular context during retrieval practice and test. Consistent with our findings, Norman et al. simulated RIF for novel episodic associations with larger context scale parameter values than when simulating RIF in semantic settings, suggesting that the requirement to try to reinstate the original context during retrieval practice may be very important.

A final methodological point to note is that in each of our experiments, we examined retrieval practice and RIF effects in one or more groups of 40 participants. With 40 participants we should be fairly confident in detecting significant retrieval practice and RIF effects of medium effect size at *p* < .05. Specifically, if we were to seek to be 80% confident of obtaining a medium effect (Cohen’s *d* = 0.5), then we would should sample 33 participants at α = .05. A comparison of the observed effect sizes in Table 1, show that we have reliably observed significant differences due to retrieval practice in all our groups, and we have obtained RIF in the majority of our groups. However, it should be noted that in some experiments, we additionally compare the relative magnitudes of retrieval practice and RIF across groups. Power considerations for comparing independent samples reveal that to be 80% confident of obtaining a medium effect (Cohen’s *d* = 0.5) at α = .05, then we would use 66 participants per group. We should therefore treat with caution nonsignificant differences between groups in the magnitude of the retrieval practice and RIF effects.

Although the current findings are highly promising for the development of human memory augmentation, there are a number of remaining challenges that must be overcome before it can become a reality. First, it should be noted that devices such as SenseCam, Google Glass, and Narrative Clip are not widely used. Their expense (which is in addition to that of a smartphone) is perhaps a major reason for the relative rarity of these devices. However, a further limitation may be the perceived social, privacy and security concerns of wearable cameras (Davies et al., 2015). We believe that attitudes to wearable cameras may change, as the use of social media continues to promote the publishing and broadcasting of one’s life, and consumers see the benefits of sharing and reviewing their memories. A barrier to common practice would be removed if consumers could use their own smartphones and new smartphone apps rather than having to buy a separate wearable camera.

Second, the full benefits of using an augmented memory device may not yet have been widely appreciated. In a critique of unthinking total capture of everything, Sellen and Whittaker (2010) have defined five benefits of augmented memory (the five Rs): recollecting (retrieving forgotten details, such as locations of lost objects, faces and names, details of meetings), reminiscing (e.g., reliving past experiences for emotional or sentimental reasons; see, e.g., Petrelli & Whittaker, 2010), retrieving (e.g., aiding in search for digital information), reflecting (e.g., abstracting behavior patterns over time to reassess one’s past experiences through new perspectives), and remembering intentions (e.g., prospective memory tasks, such as running errands, taking medication and attending appointments). Within this framework, there are clear benefits from an end-of-day review to accomplish all five benefits, and as society understands the benefits to work, health, learning and everyday life (and possibly even afterlife; Bell & Gemmell, 2009), so concerns regarding privacy may be reduced.

More recently, Gurrin et al. (2014) contrasted personal lifelogging applications from population-based lifelogging applications. They defined personal lifelogging applications as those where a single individual uses lifelogging tools to record information about him/herself primarily for his or her own benefit. Typical personal applications include the quantification of personal data (calories eaten, paces walked, cigarettes and alcohol units consumed, hours slept) often with the aim of reviewing (and potentially changing) one’s behavior (often through gamefication), memory assistance whether as part of memory rehabilitation (e.g., Allé et al., 2017; Silva et al., 2013) or to augment normal healthy memory (e.g., Finley et al., 2011; Sellen et al., 2007). The end-of-day review is particularly well placed to help with the latter applications.

By contrast, Gurrin et al. (2014) defined population-based lifelogging applications as those where the lifelogs are processed and combined to allow us to infer something about the population of users as a group. They noted three applications: the capturing and processing of procedures within a corporation to aid in understanding and improving of corporate practices, the enrichment of lifestyle data and exposure to brand names and logos to be used by market research firms, and the aggregation of individual family memories to form a shared family event. It is possible that although users may initially view their personal captured data as intensely private, they nevertheless may come to realize some advantages for sharing their data.

A further limitation for an end-of-day review is the difficulty in selecting the most relevant captured data to be represented in a sequence of images (or indeed summary video; e.g., Le, Clinch, Sas, Dingler, Henze, & Davies, 2016). One partial solution to this difficulty might be for participants to contribute actively to the selection of the reviewed material by consciously tagging certain moments throughout their days as moments to be later reviewed. If this tagged information was shared such that these conscious tags were exchanged with individuals who were in similar locations at similar times, then these population-shared data could help supplement sources of captured data and help through a type of crowd-sourcing identify suitable “highlights” for an end-of-day review.

Overall, our findings of retrieval practice are consistent with the beneficial effects of a review of captured images (e.g., Allé et al., 2017; Sellen et al., 2007; Silva et al., 2013). An end-of-day review (Finley et al., 2011) offers the user the ability to reinforce learning from the day as well as strengthen the intention for prospective actions. Finally, Davies et al. (2015) identified a number of related future uses for memory augmentation systems that could capture, process, store and display information. These also included behavior change (especially if the systems remind the user of their own and their loved ones’ attitudes to behaviors, and the system aids in realistic scheduling), learning new information (whether this be to support formal education or to support recreational study; e.g., second-language learning), supporting failing memories and advertising (being reminded to purchase items known to be needed by the user at convenient times, when there is a good opportunity to purchase these items). Interestingly, the discovery of RIF using this material offers the first evidence supporting the potential for Davies et al.’s final future use: that of selective recall, the development of retrieval practice schedules with the intention of desired attenuation of unwanted or out-dated memories.

Further work is necessary to automatically segment lifelogging data into annotated events (e.g., Doherty et al., 2011; Doherty & Smeaton, 2008; Hamm et al., 2013) and integrate these highlighted images with other captured data such as location (e.g., Kalnikaite, Sellen, Whittaker, & Kirk, 2010). In addition, further work is also needed to test the longevity of these effects. Previous studies (e.g., Experiment 1) suggest that the effects of retrieval practice (or effects of testing, Roediger & Karpicke, 2006a, 2006b) may be more long-lasting than the effects of RIF (e.g., Macrae & MacLeod, 1999), but if the practice of an end-of-day review is used habitually, then an interactive review schedule could be used to insert tagged events into the end-of-day review on subsequent days to benefit from expanded and distributed retrieval practice.

Finally, smartphone and life-logging technology offers researchers interested in the psychology of memory a more complete, more objective, more contemporaneous and more incidentally encoded record of autobiographical events to interrogate our memory capabilities than that possible to even the most committed diary writing by memory researchers (e.g., Linton, 1975; Wagenaar, 1986). It also allows carefully controlled experimental stimuli to be presented via personal devices at time intervals well beyond what is practical in the laboratory (e.g., Cortis Mack, Cinel, Davies, Harding, & Ward, 2017).

In summary, our experiments provide the first steps toward the scientific foundation for the development of an end-of-day review that could be used to augment human memory. We provide evidence for retrieval practice and RIF effects in the real world, and extend these findings to participant-generated stimuli, provide manipulations to amplify the effects of practice and RIF, and have prototyped a user interface for selecting and scheduling practice for to-be-remembered and to-be forgotten items. We believe that these findings encourage the development of technologies that capture, store, process and display selected data to be used as cues to augment human memory. The application of these findings could help further the vision of SenseCam to be used as a memory aid for both healthy and memory-impaired populations.

## Supplementary Material

10.1037/xge0000441.supp

## Figures and Tables

**Table 1 tbl1:** The Mean Proportion of Correctly Recalled Exemplars (With Standard Errors in Parentheses) for the Three Levels of the Retrieval Practice Status Across Experiments 1 to 4

Experiment	Retrieval practice status			Effect sizes (Cohen’s *d*)	
	Rp+	Nrp	Rp−	Rp	RIF
Experiment 1					
RP group: First test	.798 (.023)	.597 (.024)	.529 (.030)	**1.284**	**.465**
RP group: 7-day test	.658 (.037)	.440 (.031)	.404 (.032)	**.941**	.183
Experiment 2	.798 (.021)	.658 (.019)	.513 (.026)	**.982**	**.887**
Experiment 3A					
3 repetitions	.779 (.023)	.500 (.024)	.448 (.028)	**1.681**	**.354**
6 repetitions	.865 (.022)	.490 (.029)	.408 (.031)	**2.049**	**.500**
Experiment 3B					
1 practiced exemplar	.875 (.027)	.501 (.024)	.461 (.030)	**1.938**	.267
3 practiced exemplars	.785 (.022)	.486 (.025)	.423 (.036)	**1.930**	**.337**
5 practiced exemplars	.741 (.023)	.533 (.022)	.331 (.038)	**1.625**	**.795**
Experiment 3C					
6 exemplars	.715 (.028)	.385 (.029)	.354 (.033)	**1.574**	.172
12 exemplars	.585 (.029)	.311 (.018)	.247 (.018)	**1.590**	**.703**
Experiment 4					
Maximize remembering	.850 (.033)	.644 (.020)	.643 (.024)	**.912**	.008
Maximize forgetting	.766 (.022)	.681 (.022)	.550 (.042)	**.787**	**.545**
*Note.* The right hand pair of values show the effect sizes (Cohen’s *d*) relating to the effect of retrieval practice (Rp; the differences between RP+ and Nrp) and retrieval induced forgetting (RIF; the differences between Nrp and Rp−) in each row condition. The bold values represent effect sizes of comparisons that were significantly different from Nrp.					

**Table 2 tbl2:** Data From Experiments 5 and 6

Experiment	Remember	Neutral	Forget
Experiment 5			
Maximize Remembering	**.771** (.019)	.446 (.025)	.394 (.025)
No RP	.604 (.022)	**.479** (.023)	.471 (.026)
Maximize Forgetting	.659 (.020)	.614 (.021)	**.357** (.023)
Experiment 6			
Maximize Remembering	**.689** (.028)	.447 (.026)	.464 (.032)
No RP	.514 (.038)	**.506** (.032)	.433 (.026)
Maximize Forgetting	.653 (.025)	.639 (.029)	**.367** (.028)
*Note.* The mean proportion of correctly recalled items based on participants’ individual selections (remember, neutral, forget) for each of the three types of retrieval practice (RP) schedules (maximize remembering, no RP, maximize forgetting). For Experiment 5, the data are aggregated over the 1 RP session and 2 RP sessions groups. Values in bold contrast the combined positive and negative effects of participant-selected memory preferences and retrieval practice schedule in the interactive review. Values in parentheses represent the standard error of the mean. The maximize remembering schedule comprised of retrieval practice of three exemplars that participants chose to remember from three randomly selected categories. The maximize forgetting schedule comprised of retrieval practice of three exemplars that participants chose to remember, plus three neutral items from three randomly selected categories. The No RP are those three other categories that received no NP.			

## Footnotes

1 To obtain eight subgroups of exactly 10 participants, we scheduled additional participants anticipating some no shows. We tested in total 83 participants and we used the data from the first 10 in each group who returned to be tested.

## A The Stimuli for the University of Essex Campus Tour Used in Experiment 1

**Table:**

	**Exemplar 1**	**Exemplar 2**	**Exemplar 3**	**Exemplar 4**	**Exemplar 5**	**Exemplar 6**
**Square 4**	**Letterbox**	**Seating**	**Bank**	**Columns**	**Lamps**	**Diner**
	can hold up to 15kgs of post	originally intended for more pots to be placed	Barclays was the first bank to open on campus	were originally painted yellow	have been changed 5 years ago	has 26 items on the menu
**Garden**	**Pebbles**	**Flowers**	**Decking**	**Gazebo**	**Birdhouse**	**Stool**
	these are artificial	potted for graduation ceremonies	used to be stained in green	removed when it snows	placed specifically for robins	used to be at Wivenhoe house
**Podia**	**Speed-limit**	**Ramp**	**Gate**	**Container**	**Mirror**	**Bicycles**
	this is the slowest speed allowed on campus	one out of 8 under the podia	only accessible by Estate management	used to store gardening tools	these are cleaned every September	91% of bike users lock bikes here
**Biology Building**	**Aquarium**	**Lockers**	**Thermometer**	**Hazard**	**Laboratory**	**Sink**
	can hold up to 36 goldfish	used for staff marking	only non-digital one on campus	compulsory for all chemical labs	there are 7 in total	is disinfected after each lab session
**Teaching Centre**	**Bins**	**Lift**	**Sofas**	**Vending Machines**	**Railing**	**Monitor**
	these replaced all individuals bins in all rooms	maximum capacity of 4 persons	were upholstered for the 50th year anniversary	they are stocked at 7.30am everyday	diameter of about 6cm	controlled by the Timetabling department
**Library**	**Sculpture**	**Paternoster**	**Floorplan**	**Printer**	**Portrait**	**Dolls**
	Its name is ‘Big Cat’	1913 original lift	includes 73 subject names	most used printer in the library	of Albert Sloman—founding Vice Chancellor	donated by a Japanese diplomat
**Lake**	**Student Centre**	**Bench**	**Tree**	**Fountain**	**Artwork**	**Barbecue**
	funded by the Silberrad fund	there are 72 identical benches on campus	painted in John Constables painting	switched off when there are strong wind warnings	sculpture by John Maine	installed following a student survey
**Car Park**	**Barrier**	**Arrow**	**Chain**	**Fees**	**Tyres**	**Disabled Sign**
	only opened in case of emergencies	repainted after the summer months	have been replaced six years ago	no fees are paid at the weekend	when the carpark is full there are a total of 5600 tyres	there are 100 disabled parking spaces across campus
**Sports Centre**	**Goal Post**	**Regulations**	**Turf**	**Basketball**	**Floodlight**	**Obstacles**
	there are two sizes: adult and children	apply for all court types	will be replaced next year	least popular sports on campus	these are switched on at 4pm in Winter	these are used by the Athletics club twice a week
**Student Union**	**Pub**	**Poster**	**Fire Extinguisher**	**Speaker**	**Telephone**	**Hairdresser**
	open until 2am on Mondays	changed every fortnight	can weigh between 1 and 23kgs	there are 21 round speakers across campus	was installed in 1994	offers 3 types of hair straightening
*Note*. For each of 10 locations, there are six to-be-remembered exemplars, and for each exemplar there is an associated fictitious comment.						

## B The Mean Proportion of Correctly Recalled Exemplars for All Three Levels of the Retrieval Practice (Rp) Status for All Technology Groups in Experiment 1

**Table:**

Experiment	Rp+	Nrp	Rp−
Experiment 1			
Immediate test			
No technology—RP group	.858 (.037)	.604 (.045)	.500 (.062)
MGOK—RP group	.783 (.043)	.575 (.056)	.542 (.060)
Smartphone—RP group	.717 (.056)	.546 (.042)	.467 (.061)
Narrative clip—RP group	.833 (.037)	.663 (.045)	.608 (.056)
No technology—NRP group		.742 (.049)	
MGOK—NRP group		.604 (.033)	
Smartphone—NRP group		.615 (.028)	
Narrative Clip—NRP group		.579 (.045)	
Overall	.800 (.023)	.616 (.016)	.529 (.030)
Delayed test			
No technology—RP group	.725 (.072)	.475 (.066)	.392 (.072)
MGOK—RP group	.567 (.088)	.338 (.058)	.425 (.080)
Smartphone—RP group	.642 (.080)	.429 (.058)	.375 (.053)
Narrative clip—RP group	.700 (.050)	.517 (.064)	.425 (.052)
No technology—NRP group		.667 (.050)	
MGOK—NRP group		.473 (.051)	
Smartphone—NRP group		.479 (.051)	
Narrative clip—NRP group		.496 (.056)	
Overall	.658 (.037)	.484 (.022)	.404 (.032)
*Note.* The values in parentheses represent the standard error of the mean. MGOK = My Good Old Kodak application.			

## C The Categories and Respective Exemplars Used as Stimuli in Experiment 3A, 3B, and 3C

**Table:**

**Categories**									
**Fruits**	**Leather**	**Trees**	**Professions**	**Drinks**	**Hobbies**	**Metals**	**Weapons**	**Insects**	**Fish**
Banana	Shoes	Oak	Nurse	Vodka	Dancing	Gold	Sword	Beetle	Trout
Orange	Wallet	Palm	Lawyer	Milk	Gaming	Silver	Rifle	Cockroach	
Strawberry	Belt	Willow	Cleaner	Juice	Drawing	Aluminum	Bomb	Hornet	Herring
Lemon	Purse	Maple	Engineer	Whiskey	Knitting	Bronze	Pistol	Fly	Salmon
Pineapple	Gloves	Birch	Accountant	Rum	Biking	Mercury	Tank	Mosquito	Haddock
Raspberry	Skirt	Fir	Dentist	Gin	Stamps	Nickel	Crossbow	Grasshopper	Angler
**Grape**	**Sofa**	**Cedar**	**Manager**	**Beer**	**Travelling**	**Steel**	**Knife**	**Caterpillar**	**Tuna**
**Apricot**	**Bracelet**	**Spruce**	**Driver**	**Wine**	**Cooking**	**Copper**	**Spear**	**Worm**	**Bass**
**Kiwi**	**Saddle**	**Elm**	**Singer**	**Tea**	**Walking**	**Platinum**	**Grenade**	**Dragonfly**	**Cod**
**Mango**	**Trousers**	**Beech**	**Builder**	**Fanta**	**Fishing**	**Zinc**	**Bazooka**	**Ladybird**	**Shark**
**Watermelon**	**Boots**	**Sycamore**	**Firefighter**	**Soda**	**Shopping**	**Titanium**	**Cannon**	**Cricket**	**Clownfish**
**Cherry**	**Hat**	**Ash**	**Scientist**	**Squash**	**Movies**	**Lead**	**Missile**	**Ant**	**Goldfish**
*Note*. The six exemplars in normal font in each category were used in Experiments 3A and 3B. Experiment 3C used all 12 exemplars, including the six additional exemplars in bold font.									
